# Challenging DESs and ILs in the valorization of food waste: a case study

**DOI:** 10.3389/fchem.2023.1270221

**Published:** 2023-10-24

**Authors:** Angelica Mero, Nicholas R. Moody, Elena Husanu, Andrea Mezzetta, Felicia D’Andrea, Christian Silvio Pomelli, Nathalie Bernaert, Francesca Paradisi, Lorenzo Guazzelli

**Affiliations:** ^1^ Department of Pharmacy, Università di Pisa, Pisa, Italy; ^2^ Consorzio INSTM, Firenze, Italy; ^3^ Department of Chemistry, University of Nottingham, Nottingham, United Kingdom; ^4^ Flanders Research Institute for Agriculture, Fisheries and Food (ILVO), Technology and Food Science Unit, Melle, Belgium; ^5^ Department of Chemistry, Biochemistry and Pharmaceutical Sciences, University of Bern, Bern, Switzerland

**Keywords:** ionic liquids, deep eutetic solvent, biomass valorization, cellulose saccharification, food waste

## Abstract

In this study, the efficacy of two of the best performing green solvents for the fractionation of lignocellulosic biomass, cholinium arginate (ChArg) as biobased ionic liquid (Bio-IL) and ChCl:lactic acid (ChCl:LA, 1:10) as natural deep eutectic solvent (NADES), was investigated and compared in the pretreatment of an agri-food industry waste, apple fibers (90°C for 1 h). For the sake of comparison, 1-butyl-3-methylimidazolium acetate (BMIM OAc) as one of the best IL able to dissolve cellulose was also used. After the pretreatment, two fractions were obtained in each case. The results gathered through FTIR and TG analyses of the two materials and the subsequent DNS assay performed after enzymatic treatment led to identify ChArg as the best medium to delignify and remove waxes, present on the starting apple fibers, thus producing a material substantially enriched in cellulose (CRM). Conversely, ChCl:LA did not provide satisfactorily results using these mild conditions, while BMIM OAc showed intermediate performance probably on account of the reduced crystallinity of cellulose after the dissolution-regeneration process. To corroborate the obtained data, FTIR and TG analyses were also performed on the residues collected after the enzymatic hydrolysis. At the end of the pretreatment, ChArg was also quantitatively recovered without significant alterations.

## Introduction

The huge amount of biomass wastes and by-products generated every year by the agri-food industry is of significant concern worldwide due to detrimental environmental impacts (Gullón et al., 2020) and increasing costs of its disposal (Lin et al., 2013), but at the same time it represents a solution to the demand for low-cost biomass. In this scenario, following the circular economy concept and the “zero waste” policy, great attention has been devoted to the valorization of lignocellulosic residual biomass from the agri-food sector for the production of novel commodities, bio-compounds, bio-fuels, power and heat through biorefinery processes (Liu et al., 2012; Jablonský et al., 2018). Indeed, lignocellulosic biomasses are considered a potential solution to the major issues of depletion of fossil resources and of climate changes caused by the large-scale combustion of petroleum derived fuels. Moreover, other significant advantages related to the exploitation of lignocellulosic biomass relies in their renewability, widely distribution and easy availability. Apple pomace fibers, resulting from fruit pressing, are a major European agro-related problem and yet constitute a viable option as lignocellulosic source. In particular, Northern Europe is the top worldwide producer of apple juice and related pomace, a solid residue with a high moisture content (up to 85%) and biodegradable organic load, making apple pomace bulky with high transport costs and susceptible to microbial decomposition, and subsequent generation of foul smells, or microbial fermentation and consequent decrement of available nitrogen in the soil when the apple waste is disposed into landfills, which affects the C/N ratio (Waldbauer et al., 2017). To date, apple pomace is used as source of compost, as combustion material or as animal feed, but there is the quest for more sustainable and profitable strategy for its valorization.

Lignocellulosic waste is composed of three main biopolymers: cellulose, hemicellulose and lignin. Cellulose is a long-chain linear homopolymer consisting of glucose units covalently linked by β-(1, 4) glycosidic bonds with the disaccharides cellobiose being the fundamental repeating unit. Hydrogen bond interactions between the hydroxyl groups of glucose units creates long fibril structures. Hemicelluloses are a group of heteropolysaccharides consisting of branched and shorter chains, which are comprised of pentoses (D-xylose, L-arabinose), hexoses (D-glucose, D-galactose, D-mannose, D-rhamnose), uronic acid groups and acetyl groups in varying amounts depending on the plant species. Due to their chemical structures, cellulose and hemicellulose can be hydrolysed to monosaccharides, and subsequently converted into biofuel. Lignin is a complex heteropolymer with a non-crystalline and irregular three-dimensional structure, which is mainly composed of three primary units, namely, syringyl (S), guaiacyl (G) and *p*-hydroxyphenyl (H) units, highly relevant for green chemistry applications, as it is considered the primary renewable feedstock for the sustainable production of aromatic chemicals (Gillet et al., 2017; Ročnik et al., 2022; Žula et al., 2023). Lignin is the first barrier to the full utilisation of the polysaccharide portion of the biomass. In its native form, it covers the cellulose with a cross-linked matrix formed by hemicellulose fibers in turn connected through ester and ether linkages, contributing significantly to biomass resistance against degradation. On the other hand, lignin degradation products may form deposits on the cellulose derived from pulping processes, that hinder and inhibit enzymatic biodegradation by non-productive binding with enzymes (Schoenherr et al., 2018). In addition, the crystalline portion of cellulose is also a critical hurdle as it cannot be hydrolysable by enzymes. Therefore, a pretreatment step to increase the accessibility of polysaccharides to enzymes is necessary in an optimized biorefinery process. Nowadays, different physical (milling, extrusion, microwave, ultrasounds …), chemical (acid or alkali treatment, reductive and oxidative fractionation) or physico-chemical (supercritical fluids, steam explosion, CO_2_ explosion pretreatment, ammonia fiber expansion, and liquid hot water) fractionation processes for an efficient release of fermentable sugars have been proposed (Wang et al., 2019; Mankar et al., 2021; Agrawal et al., 2022; Kaur et al., 2023; Sharma et al., 2023). These traditional methods present several disadvantages as they generally require toxic hazardous organic solvents and harsh condition resulting in high energy consumption and raising serious concerns in terms of safety and environmental impact, native primary structure degradation, and most of all derivatization of the components during the treatment (Mankar et al., 2021). The urge to develop more sustainable and non-derivate treatment prompted the research towards the utilization of ionic liquids (ILs) and deep eutectic solvents (DESs). Indeed, these media are capable to selectively solubilize and recover one of the three components and thus allow for the fractionation of biomass to be pursued. ILs are organic salts with a melting point below 100°C which are used in biomass pretreatment to enhance enzymatic digestibility since 2006 (Dadi et al., 2006; Liu and Chen, 2006). They possess various appealing properties including negligible vapour pressure (Barulli et al., 2021), high thermal and chemical stability (Chen et al., 2022), non-flammability, wide electrochemical range (Piatti et al., 2022), and an outstanding tunability based on the wide selection of potential constituting cation-anion pairs. Above all, ILs can dissolve a wide range of materials including hydrophobic, hydrophilic, and also polymeric compounds (Hasanov et al., 2020; Azimi et al., 2022). The first studies on the lignocellulosic biomass pretreatment with ILs involve the solubilization of cellulose with imidazolium-based ILs. These reduce the crystalline portions of native cellulose improving the enzymatic hydrolysis efficiency (Brandt et al., 2013). Over time, the attention headed to different ILs able to remove lignin from biomass. This class of ILs allow for recovering a cellulose enriched material (CRM) in the first place and for re-precipitating the dissolved lignin enriched material (LRM) in the second stage. In recent years, various IL classes proven effective in the selective dissolution of lignin (Dutta et al., 2017; Hou et al., 2017). Among them, renewable cholinium amino acids-based ILs have been reported to have high levels of efficiency in the fractionation of lignocellulosic biomass due to their selective removing of lignin (Hou et al., 2012; Hou et al., 2015; An et al., 2015; Dutta et al., 2018). In particular, cholinium arginate (ChArg) showed remarkable performances in biomass pretreatment due to strong hydrogen bonding by the anion (Karton et al., 2018). Indeed, it was found to be effective in the treatment of grass lignocelluloses as well as *Eucalyptus*, resulting in significant improvements in the glucose yields in subsequent enzymatic digestion (An et al., 2015). Another class of eco-friendly solvents used successfully in the pretreatment of lignocellulosic biomass is accounted by deep eutectic solvents (DESs), a eutectic mixtures of two or more separate components acting as hydrogen bond acceptor (HBA) and hydrogen bond donors (HBD) showing a melting temperature of the eutectic point below to that of the ideal mixture (Martins et al., 2019). DESs possess some similar properties to ILs, for instance the qualification of designer solvents due to the possibility to select different HBA-HBD pair and tune their properties (Mero et al., 2023). In particular, their green and sustainable character may be increased when natural and renewable materials are used for their preparation, producing the so called natural deep eutectic solvents (NADESs) (Satlewal et al., 2018; 2019). Thanks to these attractive features (NA)DESs have been applied in several fields, such as bio-catalysis and organic chemistry (Cicco et al., 2021; Panić et al., 2021; Nolan et al., 2022; Thakur et al., 2022; Cvjetko Bubalo et al., 2023; Jesus et al., 2023), in pharmaceutical and medical research (Emami and Shayanfar, 2020; Huang et al., 2021; Oliveira et al., 2023), extraction of metal and bioactive molecules (Serna-Vázquez et al., 2021; Elahi et al., 2022; Moni Bottu et al., 2022), in development of analytical protocols (Ul Haq et al., 2022; Ullah et al., 2022), and biomass and food processing (Mamilla et al., 2019; Bjelić et al., 2020; Ling and Hadinoto, 2022; Moccia et al., 2022; Cheng et al., 2023). Related to this last application, different acidic-based DESs (Alvarez-Vasco et al., 2016; Shen et al., 2019) have been tested and cholinium chloride:lactic acid (ChCl:LA) is deemed one of the best performing DES in lignin dissolution (Alvarez-Vasco et al., 2016; Shen et al., 2019; Smink et al., 2019; 2020a; 2020b; Cronin et al., 2020; Hong et al., 2020; Oh et al., 2020; Tian et al., 2020; Morais et al., 2021). The comparison of the performance of ILs and DESs for shared applications is recently becoming a subject of the highest interest. Recently, some of us have reviewed the pros and cons of using ILs and DESs to selectively dissolve key biomass components (Afonso et al., 2023). However, in-depth knowledge of both media is the only way to make reasonable comparisons by selecting the most promising systems of each class for a specific application. In the field of biomass pretreatment, comparisons between different types of ILs (Hou et al., 2015; Dutta et al., 2017) or different types of DESs (Zhang et al., 2016; Guo et al., 2018; Thi and Lee, 2019; Grillo et al., 2021) were carried out. However, DESs and ILs were rarely compared. To the best of our knowledge, only one work reports the effect of DES choline chloride:boric acid in molar ratio 5:2, choline chloride:glycerol 1:1 and betaine:glycerol 1:1 and of the IL 1-ethyl-3-methylimidazolium acetate (EMIM OAc) in the pretreatment and subsequent enzymatic hydrolysis of microcrystalline cellulose, *Eucalyptus* dissolving pulp, shredded wheat straw and spruce saw dust (Wahlström et al., 2016). In addition, tests of enzyme stability in the presence of ILs and DESs showed that often DESs exhibit a lower inhibiting effect than ILs. Although of interest, this study calls for further investigations of the best performing state of the art systems on real lignocellulosic waste samples. Therefore, in the present work, a comparative enzymatic hydrolysis of apple pomace fibers, obtained from fruit juice production by Konings (Belgium), after pre-treatment was studied. The efficacy of the enzymatic process was assessed on fibers pretreated using mild condition (90°C, 1 h) with two of the most well-known and best performing green solvents in fractionation of lignocellulosic biomass: ChArg as biobased-IL and ChCl:LA 1:10 as NADES. For further comparison, 1-butyl-3-methylimidazolium acetate (BMIM OAc) as one of the classic IL able to dissolve cellulose was tested. The results in terms of FTIR spectra, TG analyses of the generated CRM fractions conducted before and after the enzymatic treatment were compared. Then, the LRM fractions were collected and analyzed to confirm their composition.

## Materials and methods

Choline Chloride, ChCl (98+%), L-lactic acid (85%–90% in water), were purchased by Alfa Aesar (Massachusetts, United States). l-Arginine (≥98%), acetic acid (≥99.8%), dry DMSO (99.9%) and ethanol (99.8%) were purchased from Sigma-Aldrich (Germany). Choline hydroxide in water solution (47%–50%) was purchased by TCI (Belgium), while 1-butyl-3-methylimidazolium (BMIM) methylcarbonate (98%) methanol solution was purchased from Proionic GmbH (Austria).

Cellulase from *Trichoderma reesei* (≥1 unit/mg) was purchased from Sigma Aldrich (Germany). Tris buffer (Ultra-pure), sodium hydroxide (98%), glucose (99%), sodium-potassium tartrate (99%), carboxyl methyl cellulose and dinitrosalicylic acid (98%) was purchased from Fisher Scientific (New Hampshire, United States).

Dried Apple fibers (pulp) were supplied by ILVO (Belgium). They were obtained by freeze-drying apple pomace collected after fruit juice production by Konings (Belgium). Then, the dried pomace were de-stoned, de-steal and pre-grinded and sieved (<1 mm). Before use, they were further grounded with blasting milling. These pretreatments allowed to obtain a more homogenous and manageable materials to facilitate the further processing.

### Synthesis of ILs

#### Cholinium arginate (ChArg)

The ChArg synthesis was performed as described previously by To et al. (2018). Briefly, prior to use, commercial choline hydroxide (ChOH) aqueous solution (ca. 47%–50%) was titrated with 1 M HCl solution giving the exact percent of ionic liquid in water (49.15%). Equal amounts (mol%) of ChOH and an aqueous solution of the amino acid were mixed and then the mixture was heated to 70°C and stirred for 3 h. At the end of the reaction, water was removed under reduced pressure. The correct stoichiometry and the purity of synthesized IL was verified by means of the ^1^H-NMR and ^13^C-NMR spectra registered in D_2_O and the data are in accordance with those reported in the literature (An et al., 2015).

#### 1-butyl-3-methylimidazolium acetate (BMIM OAc)

The BMIM OAc was synthesized as previously reported by Guglielmero et al. (Guglielmero et al., 2019). Briefly, at first, accurate concentration of BMIM methylcarbonate in the commercial methanol solution was determined by titration using a 1 M HCl solution. An equimolar amount of acetic acid was slowly added to the commercial methylcarbonate IL methanol solution at room temperature. Immediately after the acid addition bubbles evolution was observed due to CO_2_ formation. After 1 h, the mixture was concentrated *in vacuo* at 50°C to remove methanol and was further dried under high vacuum for 8 h. BMIM OAc was recovered as a slight yellowish oil and the ^1^H-NMR and ^13^C-NMR data are in accordance with those reported in the literature (Guglielmero et al., 2019).

### Preparation of NADES

#### ChCl:L-lactic acid 1:10

Prior to the NADES preparation, ChCl was dried under vacuum for 6 h at 80°C and commercial L-lactic acid (LA) aqueous solution (ca. 10%–15%) was titrated with 1 M NaOH solution giving the exact percent of lactic acid in water (86.3%). ChCl and lactic acid were weighted in the appropriate molar ratio (1:10) in a glass vial. The mixture was kept at room temperature and stirred with a magnetic stir bar until a homogenous colorless liquid was formed. ^1^H-NMR and ^13^C-NMR data are in accordance with those reported in the literature (Morais et al., 2021).

### Biomass treatment

#### Fibers prewashing protocol

Before treatment with ILs and NADES, dried apple fibers were prewashed to remove pectin. Removal of soluble pectin has been shown to improve the effectiveness of cellulase treatment on apple pomace (Luo and Xu, 2020). Jet milled pomace was suspended 2% (m/v) in 50 mM Tris pH 7.5. The suspension was then centrifuged at 4,500 g for 1 h and the soluble fraction was decanted. This process was repeated for a total of three times. The concentration of the reducing sugar in the wash solution was used to determine the effectiveness of the wash. The washed pomace was freeze-dried overnight or until the pomace was completely dried.

### Holocellulose content

Based upon the delignifying procedures described by Yokoyama et al. (2002), holocellulose content was determined. In a round bottom flask, 1 g of dried apple fiber after pectin wash and 20 mL water were mixed and heated with stirring at 90°C. Then, a sodium chlorite solution (20 wt%, 5 mL) and 2 mL of glacial acetic acid were added. The addition of sodium chlorite solution and glacial acetic acid was repeated at 30, 60, and 90 min after the first addition. 2 h after the first addition, the round bottom flask was cooled in a cold-water bath. Glass microfibers filter was used to filter the reaction mixture. The residue, holocellulose, was washed with hot water (3 × 200 mL) and acetone (20 mL) and dried at 105°C. The holocellulose content was quoted as wt% of the dried biomass weight (calculated as g of holocellulose/g of the dried biomass*100).

### Determination of α-cellulose content

For bleached and delignified pulp, the level of cellulose purity is obtained by R10, R18, S10, and S18 methods (Carrier et al., 2011). The values represent the pulp’s solubility in 10% and 18% NaOH solutions under specified conditions (standard ISO 692:1982). The soluble portion (%) of pulp in 10% and 18% NaOH is referred to as S10 and S18, respectively, while the residual fraction (%) is referred to as R10 and R18. It is known that a 10% NaOH solution can dissolve the degraded cellulose and hemicelluloses (S10), whereas an 18% NaOH solution dissolves the most important part of hemicelluloses (S18). The subtraction, S10-S18 (or R18-R10), is a measure of the degraded cellulose, S18 (or 100-R18) represents hemicelluloses and R10 corresponds to alfa cellulose and represents the “long-chain” cellulose content.

500 mg of the holocellulose obtained from the above reaction was placed in a 100 mL beaker and left for 30 min to allow moisture equilibrium. 40 mL NaOH (10% w/v) was added and left for 30 min 40 mL of water was added and stirred for 1 min with a glass stirring rod then left for another 29 min. The suspension was filtered with a sintered glass filter and washed with deionized water (3 × 100 mL). The residue was soaked in 1 M acetic acid (10 mL) for 5 min. It was then filtered and washed with deionized hot water (3 × 300 mL) followed by drying at 105°C. The same procedure was repeated using NaOH 18% w/v.

### Biomass treatment with ILs and NADES

#### Method A: treatment with ChArg

After pectin removing, biomass was treated with ChArg following the procedure previously described by Hou et al. (2015), An et al. (2015) and in our previous work (Husanu et al., 2020) with some modifications. The treatment was performed with milder condition than that described by the authors. Briefly, IL was heated under stirring at 90°C and then biomass samples were added with a biomass/IL ratio 1/10 (w/w) and stirred at the same temperature for 1 h. Subsequently, the suspension was cooled at room temperature and diluted with 50 mL of NaOH solution (0.1 M). The undissolved residue (cellulose enriched material, CRM-ChArg) was recovered by filtration and washed with the same basic solution and then water until neutral pH. After, the solid residue was freeze-dried for 24 h and stored in a sealed vial prior to characterization and enzymatic hydrolysis. The remaining filtrate was acidified to pH 2 with HCl (4 and 1 M) and stored at 4°C for a night to precipitate a second fraction enriched in lignin (LRM). The recovered material was separated by centrifugation and washed with the same acidic solution and then with water and finally freeze-dried. The two fractions were then analyzed by FTIR and TGA. Furthermore, the IL was recovered; after the separation of the LRM, the filtrate was basified to pH 11 (the pH of the aqueous solution of the IL) with NaOH (4 and 1 M). Water was removed by evaporation under reduced pressure. Anhydrous MeOH was added to the residue, leaving NaCl as the insoluble solid, which was removed by filtration. MeOH was evaporated under reduced pressure and the recovered IL was analyzed by ^1^H NMR spectroscopy.

#### Method B: treatment with ChCl:Lactic acid 1:10

After pectin removal, biomass was treated with ChCl:LA (1:10) following a procedure previously described by Morais et al. (2021) with slight modification. Briefly, the treatment was performed at 90°C or 120°C, for 1 h at a fixed biomass/NADES ratio 1/10 (w/w). Then, the mixture was cooled down to room temperature and a solution of ethanol-water (50:50) was added to reduce the viscosity of the system and to maintain lignin soluble. The undissolved residue enriched in cellulose (CRM-ChCl:LA) was recovered by filtration on a glass filter, washed three times with an ethanol-water (50–50) solution to remove the NADES, freeze-dried and further characterization and enzymatic treatments were performed. Finally, ice cold water was added to the filtrate for lignin precipitation (LRM). The precipitates were recovered by centrifugation, washed with water and then dried in the oven (60°C). Finally, the filtrate was evaporated under reduced pressure to recover the NADES that was analyzed by ^1^H-NMR.

#### Method C: treatment with BMIM OAc

After pectin washing, biomass was treated with BMIM OAc at 90°C for 1 h with a biomass/IL ratio 1/10 (w/w). Then, the suspension was cooled to room temperature and diluted with 50 mL of DMSO. The undissolved residue was recovered by filtration and washed with other DMSO and water. The obtained solid fraction was freeze-dried for 24 h. Then ethanol was added to the filtrate and the mixture was stirred at 60°C for 3 h. Regenerated cellulose was separated by centrifugation (a Remi R-8D centrifuge operating at 4,000 rpm max). The regenerated cellulose enriched material (CRM-BMIM OAc) was further washed with ethanol at 60°C for 3 h and again recovered by centrifugation. The washing procedure was repeated two more times. After the final separation, the material was freeze-dried for 24 h and stored in a sealed vial prior to characterization through FTIR and TG analyses and enzymatic hydrolysis.

### Enzymatic treatment: time course assay and weeklong treatment

Untreated and treated fibers with ILs and NADES were suspended 2% (m/v) in 50 mM Tris pH 7.5. Enzymes were added at a concentration of 1 mg/mL, 30 mg of fibers and 15 mg of enzyme in 15 mL of solution. Enzyme and fibers mixes were incubated at 30°C with shaking at 200 rpm for up to 1 week, in triplicate. Such timescale has been utilised in other studies to allow observation of effective degradation of the fibers and release of monomeric sugars, enzymatic treatment of CMC run for long yielded full degradation (Gao et al., 2014). Reducing sugar concentration was evaluated by the dinitrosalicylic acid (DNS) assay at different times during the weeklong treatment. The mass of the fibers was measured, and their conditions were recorded with a picture prior to enzymatic hydrolysis. At the end of the treatment, after 1 week, a DNS assay was performed on the soluble fraction to determine the soluble reducing sugar and the conditions of the fibers were recorded with another picture. The soluble fraction was decanted, and freeze dried overnight, the mass loss of the insoluble fibers was then calculated. Enzymatic treatment of fibers was performed in triplicate. Statistical significance of change in mass loss was determined by Kruskal–Wallis test (Graphpad).

### DNS assay: reducing sugar assay

The effectiveness of the enzyme treatment and fibers washing was evaluated by determining the concentration of reducing sugar *via* the DNS assay (Ghose, 1987). Briefly, 0.5 mL of the sugar containing solution was combined with 0.5 mL of DNS solution and then heated to 100°C for 5 min in a hot block. The solution was then chilled on ice to room temperature. The absorbance was measured at 540 nm, blanked with DNS buffer with water. The calibration curve was performed with glucose. Statistical significance of change in mass loss was determined by Kruskal–Wallis test (Graphpad).

### Inhibition assays

The sensitivity of *T. reesi* cellulase to ILs and NADES was determined. Degradation of carboxymethyl cellulose (CMC) by cellulase in the presence of several concentrations of ILs and DESs was performed. Samples of CMC assay were taken over regular intervals (every 5 min) over a 25 min period, and quenched with DNS solution. Reducing sugar was evaluated with the DNS assay, linear regression was used to determine the initial rates. Initial rates were plotted against inhibitor. IC_50_ was determined by Equation (1):

y=B+T−B1+T−B0.5×B−1×IC50^H (1)

where T is the top plateau of the *y*-axis, B is the bottom plateau of the *y*-axis, IC_50_ is the concentration at halfway between the plateaus, H is the Hill Slope.

### NMR spectroscopy

The ^1^H-NMR spectra were recorded in D_2_O, DMSO d_6_, CDCl_3_ or in MeOD on a Bruker 400 MHz NMR spectrometer at 25°C. ^13^C-NMR spectra were recorded at 100 MHz. The samples were prepared in 5 mm tubes. Chemical shifts (ppm) are referenced to the either residual D_2_O (δ_H_ 4.79), DMSO (δ_H_ 2.50, δ_C_ 39.5), CDCl_3_ (δ_H_ 7.26, δ_C_ 77.0) or MeOD (δ_H_ 3.31, δ_C_ 49.0).

### Fourier transform infrared spectroscopy

The ATR-Fourier transform infrared spectroscopy (FTIR) spectra were recorded with an Agilent Technologies IR Cary 660 FTIR spectrophotometer using a macro-ATR accessory, a Diamond crystal. The spectra were measured in the range from 4,000 to 600 cm^−1^ with 32 scans both for samples and background, measured first to eliminate the moisture and CO_2_ from the samples.

### Thermogravimetric analysis

Thermogravimetric analyses (TGA) were carried out using a TA Instruments Q500 TGA (weighing Precision ±0.01%, sensitivity 0.1 mg, baseline dynamic drift <50 mg). TG measurements were performed heating 15–20 mg of each sample at a rate of 10°C/min, from 30°C to 800°C under nitrogen flow (80 mL/min) in a platinum crucible. The instrument was calibrated using weight standards (1 g, 500 mg and 100 mg) and the temperature calibration was performed using curie point of nickel standard. All the standards were supplied by TA Instruments Inc. TGA experiments were carried out in triplicate for each sample.

## Results and discussion

In this study, dried apple fibers have been taken into account as a lignocellulosic waste, exploitable as a sustainable alternative to fossil fuel derived material since it can be considered as a source of cellulose that can be hydrolyzed into monosaccharides, and subsequently converted into biofuel. For the pretreatment of this biomass waste, two of the best performing systems reported to date, based on the same cholinium cation, ChArg as bio-based IL and ChCl:LA (1:10 M ratio) as NADES, were selected. Both green solvents are praised for their ability to dissolve selectively the lignin allowing the physical recovery of the undissolved solid material enriched in cellulose (CRM) and the following precipitation of the dissolved lignin enriched material (LRM). For further comparison, the effect of BMIM OAc, which is by far the most investigated IL capable of dissolving cellulose, was also tested on this agri-food industrial waste.

### Jet milled process

**FIGURE 1:**
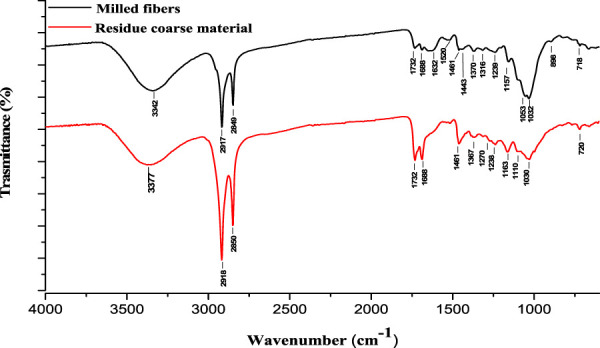
FTIR spectra of milled fibers and residue coarse material after jet milling process.

**FIGURE 2:**
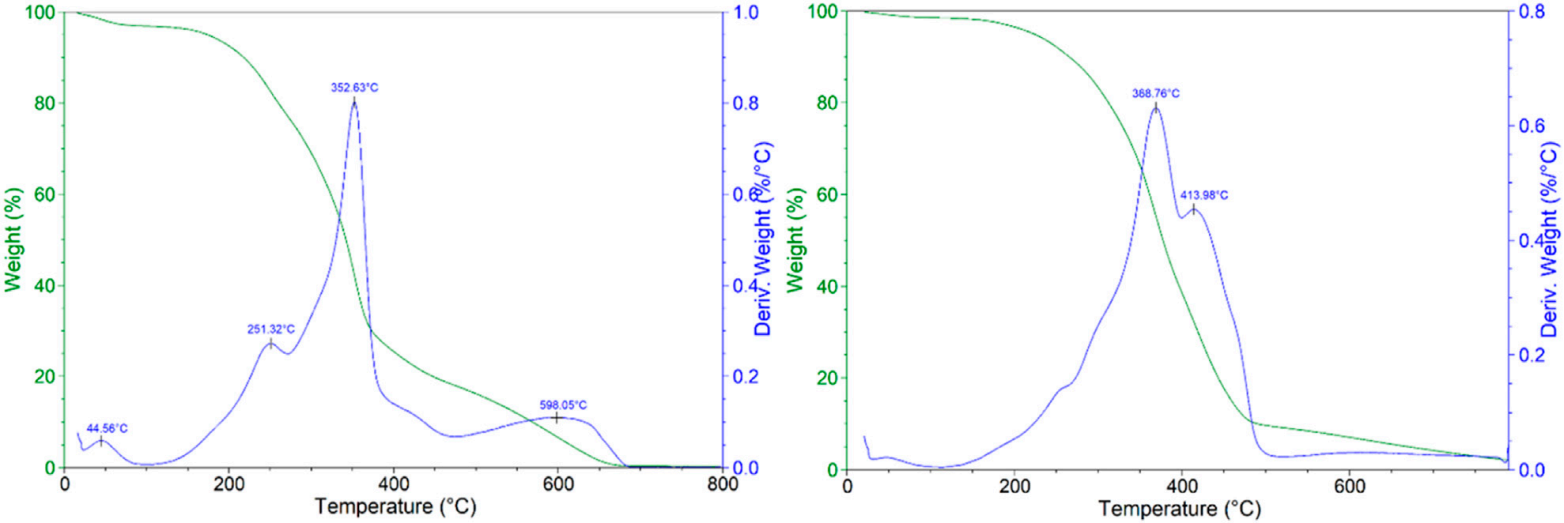
Thermograms of Milled fibers (left) and of Residue Coarse Material (right).

Before the pretreatment, apple pomace fibers were homogenized through a jet milled process. Two fractions with different particle size were obtained: “Milled fibers” with a single volume distribution in the <10 μm range and “Residue coarse material” with 2 main volume distributions showing very different dimensions (from 32.457 to 1,184.205 μm). The volume distributions are shown in Supplementary Figure S1 and Supplementary Table S1. To ascertain their composition the two materials were characterized by FTIR (Figure 1) and TG analyses (Figure 2).

The FTIR spectra showed clear differences between the two fractions. The milled fiber fraction displayed all the characteristic absorption bands attributable to the three main components of the lignocellulosic material (cellulose, hemicellulose and lignin) (Sim et al., 2012; Lupoi et al., 2014; Acquah et al., 2016). Indeed, within the fingerprint region, the bands at 898 cm^-1^ and the range between 1,200 and 1,000 cm^-1^ with a maximum value at 1,032 cm^-1^ stemmed from the vibration of β-glycosidic C-H deformation with a ring vibration contribution (hexoses/pentose) characteristic of glycosidic bonds and the C-O-C ring vibrational stretching modes of cellulose/hemicellulose glycosidic linkages. The band at 1,239 cm^-1^ is related to C-O stretching of syringyl rings, C-O stretching in lignin and hemicellulose and OH-plane deformation. The bands at 1,370 and 1,316 cm^-1^ derive from CH_2_ symmetrical vibration of cellulose/hemicellulose and CH_2_ wagging of cellulose. Instead, the bands at 1,443, 1,520 and 1,632 cm^-1^ are attributed to lignin fraction. They correspond to C=C deformation modes and stretching vibrations of lignin aromatic rings, respectively. Then, the band at 1,732 cm^-1^ is attributed to C=O stretching of acetyl and uronic ester groups of hemicellulose or the ester groups of ferulic and *p*-coumaric acids of lignin. Outside the fingerprint region, the bands occurring at 2,917–2,849 cm^-1^ are associated with the stretching of methyl and methylene groups in hemicellulose/cellulose along with the aromatic ring vibration in lignin, while the broad band in the range between 3,500 and 3,000 cm^-1^ with a maximum at 3,342 cm^-1^ is assigned to all the vibrations of the hydrogen bond of the O-H group. In addition, the FTIR spectrum could be representative also of pectin, another component typically present in apples. Pectins are another family of high molecular weight polysaccharides constituted mainly of esterified α-(1→4)-d-galacturonic acid residues, organized in a linear backbone. The linear structure of pectin is partly interrupted by (1,2)-linked side-chains consisting of l-rhamnose residues and some other neutral sugar (xylose, galactose and arabinose). Compared to cellulose, pectin displays a branched and more complex structure. In addition, the galacturonic acid units could be methyl esterified providing gelling properties (Cárdenas-Pérez et al., 2018). However, the FTIR spectrum is very similar to cellulosic materials and we can only assume the presence of pectin considering the nature of the studied biomass. Indeed, pectins characteristics bands are detected around 3,400, 2,930, 1,732, 1,460, 1,377 cm^-1^ and in the range of 950–1,200 cm^-1^ and they are related to the stretching of OH groups, CH stretching vibration, C=O stretching, CH_2_ scissoring and OH bending vibration and C–O–C stretching, respectively (Mishra et al., 2008; Zouambia et al., 2017; Hasanah et al., 2019).

On the other hand, the residue coarse material (Figure 1, bottom) did not show the characteristic bands related to polysaccharides. This fraction appeared enriched in waxes. Like most aerial parts of the terrestrial plants and fruits, apple presents an outer hydrophobic epicuticular wax layer. It is deposited in and on the cutin matrix and it serves as a shield with barrier properties against environmental stresses (i.e., wind, temperature, drought and absorption of fertilizers, growth regulators, fungicides, insecticides and herbicides, infection by plant pathogens, mechanical damage, abrasions) also after harvest during storage (Veraverbeke et al., 2001). Moreover, it provides water repellence and prevents moisture loss due to uncontrolled non-stomatal long-term storage, and loss of organic and inorganic compounds by leaching (Ibrahim J. A. et al., 2020). The epicuticular wax layer of apples is mainly composed by a mixture of long-chain hydrocarbons as major components and other aliphatic compounds like fatty acids, aldehydes, primary and secondary alcohols, ketones, and alkyl esters (Verardo et al., 2003). The FTIR spectrum of the residue coarse material displayed two very intense bands at 2,918 and 2,850 cm^-1^ that correspond to asymmetric and symmetric stretching vibration of methyl or methylene C-H of saturated aliphatic compounds. The strong methylene/methyl bending vibration at 1,461 and the weaker band at 720 cm^-1^ (methylene rocking vibration) are also indicative of a long-chain linear aliphatic structure. Finally, the bands at 1732 and 1,688 cm^-1^ are distinctive of C=O stretching of esters and/or alkyl carbonates and of carbonyl of conjugated aldehydes and ketones, respectively (Verardo et al., 2003; Ibrahim J. A. et al., 2020).

The very intense bands at 2,917 and 2,849 cm^-1^ of the milled fibers fraction, along with the weaker bands at 1,688, 1,461 and 718 cm^-1^ suggests the presence of waxes also in this fraction.

Based on these observations, the ideal pretreatment solvent should be able to separate both lignin and waxes components to obtain the desired cellulose enriched fraction. The thermogravimetric analyses also suggested a different composition of the two fractions. The milled fibers showed the four major typical degradation steps of the lignocellulosic biomass pyrolysis: moisture evaporation below 150°C, main mass loss in the temperature range 300°C–400°C with a *T*_peak_ at 353°C with a lower degradation step at 251°C attributable to cellulose and hemicellulose portions, respectively, and a long tail at high temperature with a maximum around of 598°C ascribable to lignin degradation (Manara et al., 2014; Senneca et al., 2020). However, the direct correlation to the mass loss for lignin is very difficult since its degradation occurs in a wide temperature range. Conversely, the residue coarse material displayed only two main degradation steps at 367°C and 414°C. The thermogravimetric analysis of different waxes reported in literature show a rather wide range for the degradation temperatures which are strictly related to their composition (Craig et al., 1971; Al-Sammerrai, 1985). Therefore, this technique is unreliable for getting clear information on the composition.

Summarizing, the residue coarse material is meanly characterized by waxes, while the milled fibers, although presenting the bands of waxes, are clearly composed by higher amounts of the three components of a lignocellulosic biomass: cellulose, hemicellulose and lignin. Therefore, to compare the ability of ILs/NADES in the fractionation of agro-industrial biomass, milled fibers were selected for further treatments and subsequent enzymatic digestion.

### Fibers prewashing protocol

At first, the biomass was subjected to a prewashing to remove pectin. After the washing procedure a mass loss of 15% was observed. The depectinized residue was characterized by FTIR (Supplementary Figure S2) and TGA (Supplementary Figure S3) analyses. No substantial differences were expected based on the data available which were mentioned above. Indeed, the FTIR spectra of biomass composed of cellulose/hemicellulose or pectin (Zouambia et al., 2017; Hasanah et al., 2019) present very similar bands. Therefore, no significant differences were registered between the untreated milled fibers and the washed biomass. The same observations hold true for the thermogravimetric analyses.

After the washing protocol, cellulose content equal to 59% was obtained. From the delignified biomass, values of 55%, 42% and 13% for α-cellulose, hemicellulose and degraded cellulose content respectively, were determined.

### Biomass treatment with ILs and NADES

The washed fibers were treated with ChArg, one of the best performing bio-based and non-toxic IL for lignocellulosic biomass fractionation. This IL was already successfully applied by An et al. in the pretreatment of different biomasses such as rice straw, sugarcane bagasse, corncob, wheat straw, pine and *Eucalyptus* (An et al., 2015). Besides ChArg, other cholinium amino acids ionic liquids have been investigated and efficiently used for the pretreatment of rice straw by Hou et al. (2012). A similar procedure was here performed with some modifications. More in details, milder conditions and higher biomass loading were tested than the previous biomasses. Indeed, the treatment was carried out using 1/10 biomass/IL ratio for 1 h at 90°C instead of 1/15 biomass/IL ratio for 12 h at 90°C. Taking into account the different nature of the investigated biomass, that shows a lower lignin content compared to wood or chestnut shells, milder conditions (drastic reduction of treatment time, 1 h vs*.* 12–16 h) and higher biomass loading (10% vs*.* 5%–6%) were selected to ameliorate the greenness of the process, reducing the energy consumption and the amount of employed solvent. Then, a solution of NaOH (0.1 M) was added to dilute, the mixture was filtered, and the undissolved residue enriched in cellulose (CRM-ChArg) was recovered. A 33% of CRM was achieved this way (Table 1). The collected filtrate was acidified to precipitate the dissolved lignin fraction (30 wt%, Table 1), subsequently recovered by centrifugation. At the end of the process the IL was easily recovered in satisfactory amount (98%) and purity (Supplementary Figure S4), underling the sustainability of the developed treatment.

**TABLE 1:** Yields of CRM ad LRM fractions obtained with ILs and NADES.

Green solvent	CRM (weight%)	LRM (weight%)
ChArg	33	30
ChCl:LA 1:10 (90°C)	76	3
BMIM OAC	48	43
ChCl:LA 1:10 (120°C)	68	5

At the same time, ChCl:LA 1:10, the most promising NADES to pretreat lignocellulosic biomass was tested. The biomass was treated in the same conditions of ChArg,1/10 biomass/NADES ratio, 90°C and 1 h, instead of 130°C for 1–8 h, reported in literature by Morais et al. (2021) The undissolved residue (76 wt%, Table 1) was recovered by filtration after the addition of an ethanol:water (50:50) solution. The lignin enriched material (LRM-ChCl:LA) was precipitated adding ice cold water to the filtrate and was recovered by centrifugation. This way only 3% of LRM was obtained (Table 1). The recovery of the NADES was attempted evaporating the supernatant obtained after LRM-ChCl:LA separation under reduced pressure. A slight increase of the known ester side product derived by the reaction between the hydroxyl group of choline chloride and lactic acid, typically observed for choline chloride acid-based DES (Rodriguez Rodriguez et al., 2019) (signals at 2.99 ppm, 3.53–3.55 ppm and 4.38–4.46 ppm), was detected in the recovered NADES (Supplementary Figure S5). Furthermore, signals not related to either lactic acid or ChCl, but derived from the lactide obtained by the condensation of two molecules of lactic acid were detectable (signals at 4.86–4.91 ppm and 1.20–1.30 ppm) (Morais et al., 2021), although they are present also in the spectrum of the freshly prepared NADES.

**FIGURE 3:**
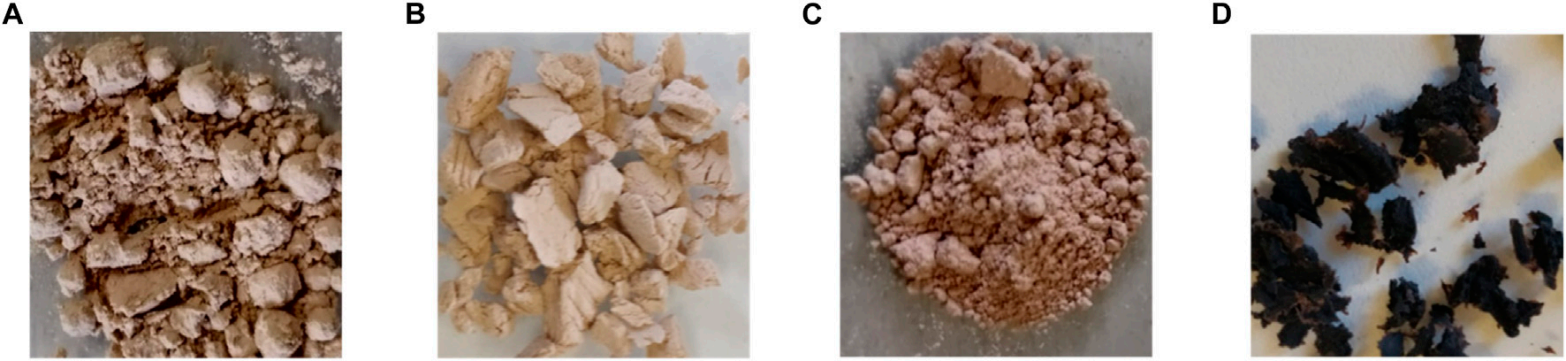
Untreated Milled fibers after pectin washing **(A)**; CRM-ChArg **(B)**; CRM-ChCl:Lactic acid 1:10 **(C)**; CRM-BMIM OAc **(D)**.

Finally, for the sake of comparison, the biomass was treated with BMIM OAc, one of the best performing IL in cellulose dissolution. In contrast to ChArg and ChCl:LA, which facilitate the following enzymatic digestion by extracting lignin from the biomass, BMIM OAc can promote the enzymatic hydrolysis by reducing the crystallinity of cellulose during the dissolution process (Yu et al., 2018). In this comparative study, the same conditions in terms of time, temperature and biomass loading were tested. After treatment, DMSO was added to reduce viscosity and filter the undissolved residue (43 wt%, Table 1). Then, the dissolved cellulose (CRM-BMIM OAc) was regenerated with ethanol and 48 wt% of material was recovered (Table 1). Figure 3 depicts the visual aspect of the untreated fibers and the cellulose enriched materials obtained from the ChArg, ChCl:LA and BMIM OAc treatment, respectively. A dark brown and tough material was observed for the biomass treated with BMIM OAc, while with the cholinium-based green solvents light brown and powdery materials, very similar to the untreated fibers were achieved. The darkening of the recovered solid from the treatment with BMIMOAc could be ascribable, as widely explain in the work of Clough et al. (2015), to the use of dialkylimidazolium ionic liquids incorporating carboxylate anions, that react with cellulose and low molecular weight sugars to generate a series of intermediates leading eventually to cations with a hydroxymethyl substituent at the C2 position of the ring. Hence, the formation of these degradation products, even in very low amounts, can lead to dark residues.

**FIGURE 4:**
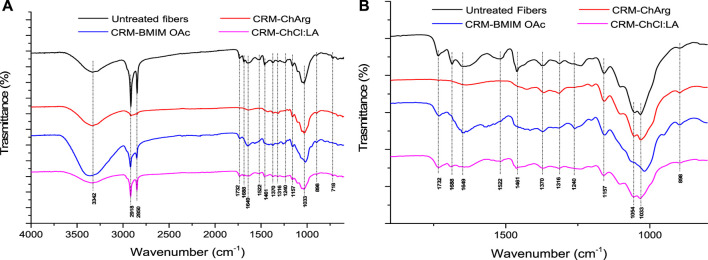
Comparison of FTIR spectra of untreated fibers and CRM-ChArg, CRM-ChCl:LA and CRM-BMIM OAc in the range 600–4,000 cm^-1^ **(A)** and the enlargement of the range 600–1800 cm^-1^ **(B)**.

All the recovered fractions were characterized by FTIR and TG analyses. The comparison of FTIR spectra of the cellulose enriched materials is reported in Figure 4.

From the FTIR spectra, it is evident that a substantially cellulose enriched material was obtained only when ChArg was used as extracting medium. All the characteristic bands of hemicellulose and lignin (1,240, 1,522, 1732 cm^-1^) and waxes (1,461, 1,688, 2,850 and 2,918 cm^-1^) disappeared or are significantly reduced (2,850 and 2,918 cm^-1^). In case of CRM-ChCl:LA, only a slight decrease of the bands attributable to lignin was noticeable, while the typical bands of waxes were still present and very intense, especially those at 2,850 and 2,918 cm^-1^. The fraction recovered after the treatment with BMIM OAc presented an intermediate situation between those obtained from the cholinium based-green solvents. In particular, a visible decrease of the bands attributable to waxes was detected. These findings are confirmed by TG analyses (Supplementary Figure S6). The CRM-ChArg showed a main degradation step at 365°C confirming that the bio-based IL did not dissolve and modify the cellulosic portion. Instead, the CRM-ChCl:LA remained very similar to the untreated sample with a slight decrease in the *T*_peak_ ascribable to cellulose degradation (339°C vs. 355°C of untreated fibers). As reported, BMIM OAc was not selective in the dissolution of cellulose (Brandt et al., 2013). Indeed, the regenerated material still showed three main steps of degradation at 231, 300°C and 435°C, ascribable to hemicellulose, cellulose and lignin respectively.

The results obtained with the NADES were in agreement with those reported by Morais et al. (2021). The authors observed that higher temperature and time were needed to extract lignin from *Eucalyptus globulus* wood. To verify the effect of harsher conditions, the treatment was performed at 120°C for 1 h. It is already reported that NADES ChCl:LA is stable up to 130.02°C and, hence, it can be employed at this temperature (Škulcová and Jablonský, 2018). A slightly darker powdery material (Supplementary Figure S7) was obtained, but no significant improvements were noted from FTIR spectra and TGA analyses (Supplementary Figure S8, S9). In agreement to what reported by Morais et al. (2021), the increase of the temperature of the extraction step caused in the present case the intensification of the band at 1732 cm^-1^. This band is related to the unconjugated C=O bonds of ester groups that could derive from the esterification of cellulose hydroxyl groups with lactic acid, a side reaction previously reported. These negative results, related to the experiments performed at 120°C, strengthened the suitability to the selected milder conditions.

### Enzymatic treatment of fibers

**FIGURE 5:**
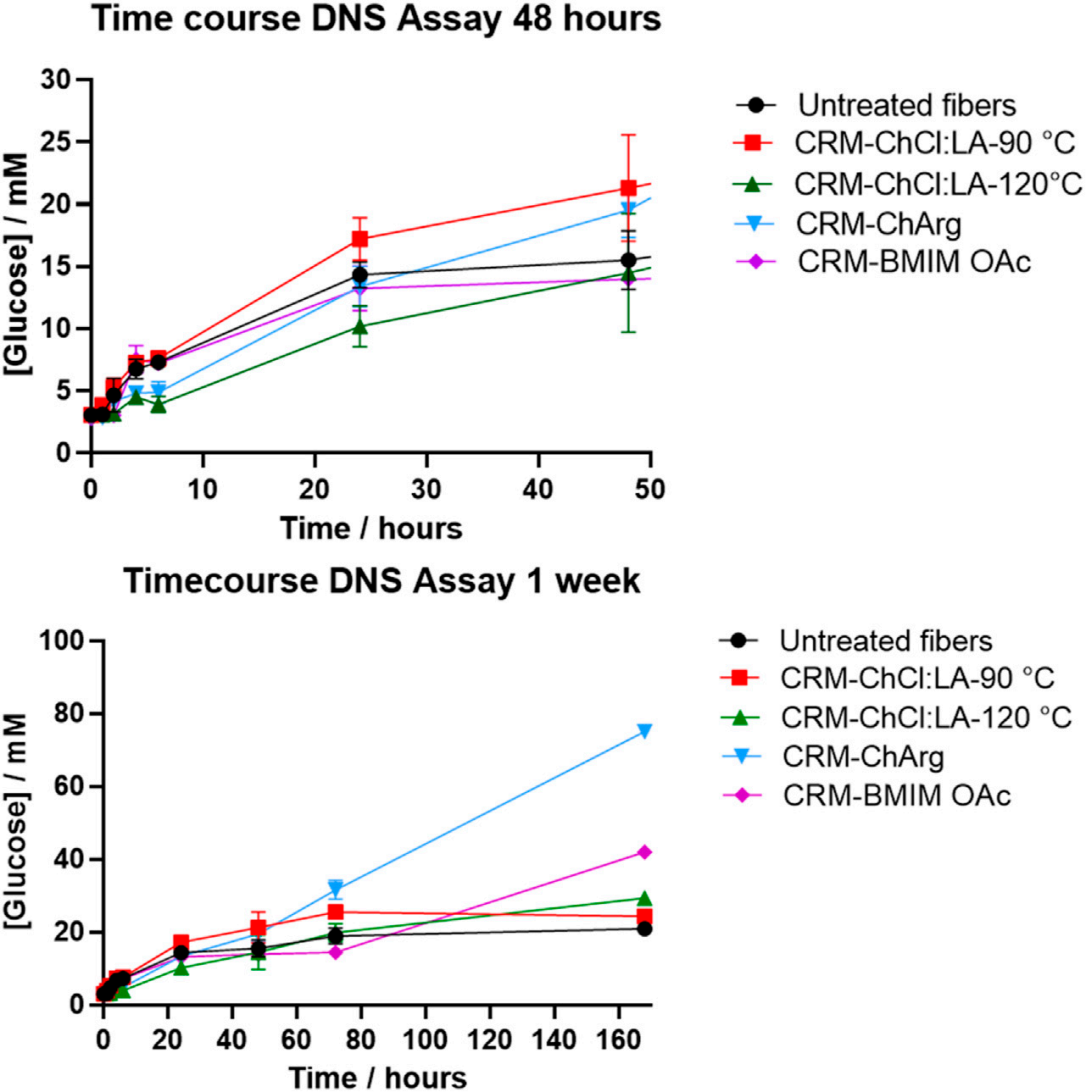
Soluble reducing sugar concentration against time as an evaluation of cellulase activity. The first graph is an enlargement of the initial set of timepoints. Treatment with ChArg was shown to be statistically significant (*p* = 0.0041)

**FIGURE 6:**
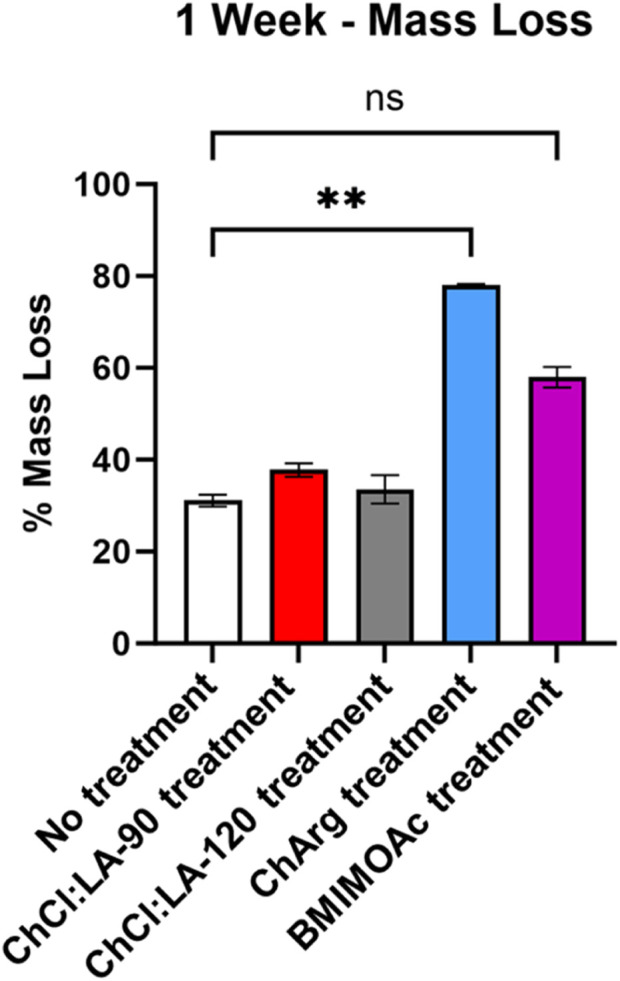
*Summary of weeklong enzyme degradation of fibers pretreated with ILs and NADES*. *Treatment with ChArg was shown to be statistically significant (p = 0.0076*).

Pretreated fibers with ILs and NADES were treated with a broad spectrum cellulase enzyme mix, and untreated fibers were used as a control. A weeklong digestion was used to evaluate the effectiveness of the ILs and NADES pretreatment. Furthermore, a time course DNS assay was performed to evaluate the initial rate of the enzyme activity. CRM-ChCl:LA-90°C shows the highest initial rate, producing the most soluble sugar in the first 48 h (Figure 5). The lowest initial rate was observed for fibers pretreated with ChArg. However, after 1 week CRM-ChArg showed the greatest amount of soluble reducing sugar and the greatest mass loss, followed by CRM-BMIM OAc and last CRM-ChCl:LA (either for treatment performed at 90°C and 120°C) (Figures 5, 6). The fractions obtained with both CRM-ChCl:LA pretreatments displayed results similar to the control sample (untreated fibers) after 1 week of incubation with enzyme (Figure 6). This would suggest that the pretreatment with ChArg leads to greater removal of lignin and consequent increased cellulose accessibility, followed by the pretreatment with BMIMOAc and ChCl:LA. These data are in agreement with the FTIR and TGA analyses of the CRM-fractions subjected to the enzymatic treatment. Indeed, they confirmed the obtainment of a fraction enriched in cellulose only after ChArg pretreatment.

### Inhibition assays

**FIGURE 7:**
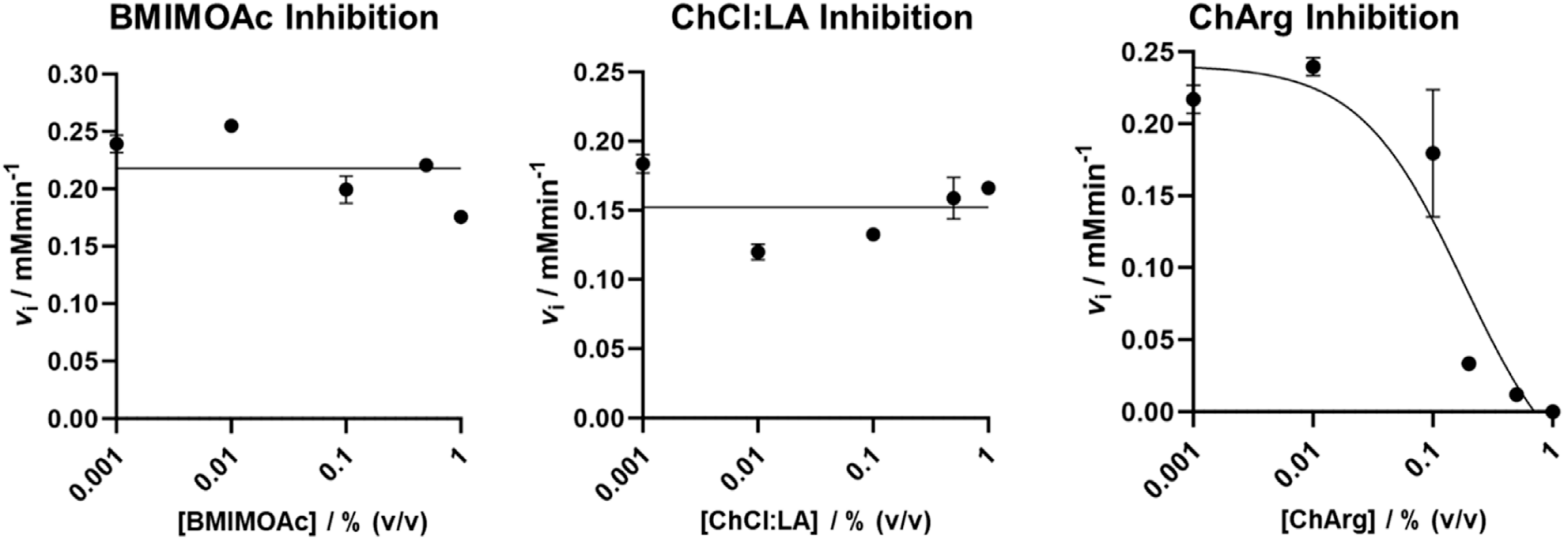
Cellulase IL and NADES inhibition assay results.

Cellulase inhibition in the presence of investigated ILs and NADES was performed to evaluate if residual traces of these solvents in the CRM fractions subjected to the enzymatic treatment could alter the results of the hydrolysis. Cellulase inhibition was not observed in the presence of BMIM OAc and of ChCl:LA NADES over the concentration range analyzed in this study (0%–1% v/v). Conversely, a slight inhibition of cellulase was detected in the presence of ChArg, showing an IC_50_ of 0.18% ± 0.02% (v/v) (Figure 7). This indicated that the rate of the degradation of fibers treated with ChArg may be reduced or even halted by its presence.

Therefore, the previous results of the enzymatic treatment demonstrate the correct removal of ChArg from the fibers after the pretreatment process. Indeed, CRM-ChArg showed the highest amount of reducing sugar after the weeklong enzymatic treatment thus suggesting that, in addition to delignifying and removing waxes from the fibers, no inhibition of the enzyme occurred.

#### Characterization of insoluble residues after enzymatic treatment

**FIGURE 8:**
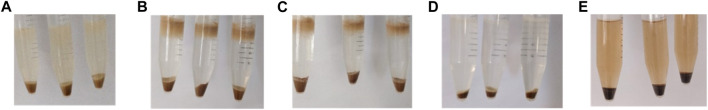
Examples of insoluble residues after 1 week of treatment by cellulase enzymes, pretreated and control fibers. **(A)** Untreated fibers, **(B)** ChCl:LA-90°C treatment, **(C)** ChCl:LA-120°C treatment, **(D)** ChArg treatment, **(E)** BMIMOAc treatment.

As a further confirmation of the enzymatic results, all residues obtained after this treatment were recovered and freeze dried (Figure 8). Then, they were again analyzed through FTIR and TGA techniques (Supplementary Figures S10, S11).

The spectra acquired from the enzymatic residues after the pretreatment with an IL (ChArg and BMIM OAc) showed a significant reduction of the bands around 1,030 cm^-1^ while the corresponding thermogravimetric curves displayed a drastic lowering of the mass loss relative to the step ascribable to the degradation of the cellulosic portion in the range 300°C–400°C. At the same time, the characteristic bands of lignin became clearly detectable. Less noticeable differences have been observed especially from FTIR analyses for the residues after the enzymatic treating of untreated fibers and the fibers pretreated with NADES. These findings confirm the results of the enzymatic hydrolysis and of the DNS assay.

### Characterization of LRM fractions

**FIGURE 9:**
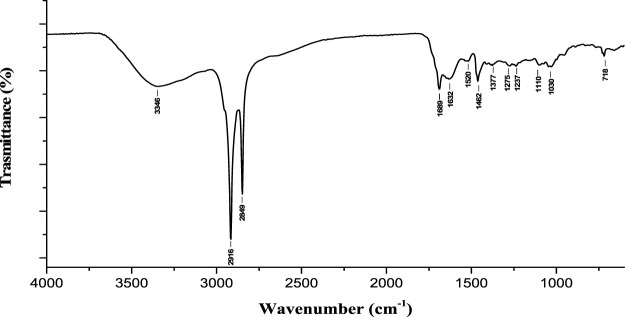
FTIR of LRM fraction after ChArg treatment (90°C, 1 h).

**FIGURE 10:**
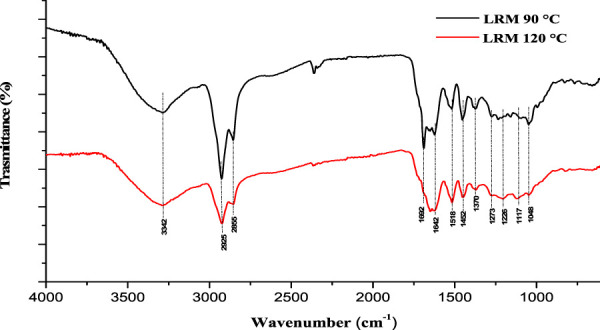
FTIR of LRM fractions after ChCl:LA treatment carried out at 90°C and 120°C.

Finally, the lignin enriched fractions (LRM) recovered by the treatment with ChArg and ChCl:LA were also analyzed to confirm their composition. Indeed, a full valorization of the starting biomass and the reutilization of all the recovered fractions are obviously another potentially attractive gain of the developed pretreatment strategy. The FTIR spectra of LRM-ChArg (30 wt%, Table 1) and LRM-ChCl:LA obtained from the treatment at 90°C (3 wt%, Table 1) and 120°C (5 wt%, Table 1) are reported in Figures 9, 10, while the corresponding thermograms are shown in Supplementary Figure S12 (see Supplementary Material).

The characterization of LRM-ChArg indicated the presence of a lignin portion but the fraction was clearly enriched in waxes. To further demonstrate that the material was composed by both lignin and waxes, it has been subjected to extractions with hexane (to dissolve waxes) and DMSO (to dissolve lignin). The undissolved residues have been recovered by filtration, dried and reanalyzed by FTIR (Supplementary Figure S13) while the filtrates were evaporated under reduced pressure to remove the solvents and their composition was analyzed by ^1^H-NMR (Supplementary Figures S14, S15). The FTIR spectrum of the undissolved residue after the extraction with hexane exhibited the evident decreasing of the bands at 2,920 and 2,850 cm^-1^ along with the disappearance of the bands at 1,688, 1,461 and 718 cm^-1^ typical of a sample of waxes. At the same time the ^1^H-NMR spectrum obtained evaporating the respective filtrate reveals all the characteristics signals of waxes (Verardo et al., 2003), including the very intense signal at 1.20–1.40 ppm of methylene groups of the long chains of hydrocarbons and the other aliphatic compounds near the characteristic signal centered at 0.85 ppm of terminal methyl groups. Ester compounds were also present as evidenced by the unresolved signals at 1.50–1.75 (C*H*_*2*_ in β-position with respect to the ester functionality), at 2.26–2.44 ppm (C*H*_*2*_ in α-position) and at 4.00–4.10 ppm (C*H*_*2*_ of the alcoholic part of the ester). Finally, alcoholic and aldehydic compounds were detected by the signals at 3.60–3.70 and 9.75–9.80 ppm, respectively. The signals at 1.96–2.12 ppm and 5.22–5.45 ppm highlighted the presence of unsaturations. On the other hand, the ^1^H-NMR spectrum of the extracted fraction with DMSO showed the presence of aromatic protons abundant in lignin samples (Supplementary Figure S15) (Mainka et al., 2015; Korbag and Mohamed Saleh, 2016). These data confirmed the capability of ChArg to dissolve and extract not only the lignin portion, but also waxes. A previous study by To et al. (2018) where ChArg was employed for the pretreatment of algal biomass, showed the ability of this bio-IL to extract lipids leaving behind a carbohydrate rich solid, further subjected to enzymatic hydrolysis. It is worth stressing that, in our study the extraction was performed at higher biomass loading and shorter time than that reported in literature for removing lignin, highlighting the sustainability of the process.

The LRM fractions recovered from the NADES, despite the limited amounts (3 wt% at 90°C and 5 wt% at 120°C), were, instead, clearly enriched in lignin, especially the one obtained at 120°C. This sample displayed the bands at 1,226 and 1,273 cm^-1^ corresponding to the C-O stretching of syringyl rings and guayacil ring breathing in lignin, respectively (Tofani et al., 2020). The recovery of low amounts of lignin using ChCl:LA could be related to structural transformations of lignin occurred during the treatment. Indeed, as reported by Shen et al. (2020) part of the lignin fraction could be depolymerized into small fragments (through the cleavage of C−O and C−C bonds in the lignin macromolecule, dehydration and acylation of OH groups in the side chain of lignin) and cannot be precipitated as regenerated lignin after the DES pretreatment.

## Conclusion

In conclusion, despite the results reported in the literature for the pretreatment of lignocellulosic biomass with ChCl:LA NADES, in the present case non-satisfactorily results were obtained. Higher temperature and longer extraction time are likely needed to reach the delignification of the samples. The results obtained in here could be also ascribable to the presence of waxes in the samples. However, when the temperature was increased, esterification of cellulose occurred. The recovery of the solvent at the end of the process was more complicated with the NADES than with the bio-IL, ChArg. As reported by Morais et al. (2021), extending time and increasing the temperature of the extraction process affect the quality of the recovered NADES. Indeed, the decrease of the integrals of lactic acid, the increase of the signals assigned to the lactide and of the signals of the ester formed between the hydroxyl group of choline chloride and lactic acid were observed. The most positive feature of the NADES pretreatment was the selectivity towards the extraction of lignin over waxes, although very small amounts of lignin were recovered. Overall, from this comparative study, ChArg appears as the most promising green solvent for the pretreatment of apple fibers in view of the subsequent enzymatic hydrolysis. In addition, this bio-IL allows for removing and easily recovering also waxes that are present in the apple fibers. Finally, unlike the NADES, the bio-IL was quantitatively recovered without significant alterations at the end of the process. The bio-IL outperformed also the results obtained using BMIM OAc, demonstrating that the removal of lignin is the crucial factor that influences the enzymatic treatment more than the cellulose crystallinity reduction.

## Data Availability

The original contributions presented in the study are included in the article/Supplementary Material, further inquiries can be directed to the corresponding author.
