# Supplementary material for: Challenging DESs and ILs in the valorization of food waste: a case study
Source: Front Chem. 2023 Oct 24;11:1270221. doi: 10.3389/fchem.2023.1270221 (PMC10628488; doi:10.3389/fchem.2023.1270221)
Supplement: Supplementary file 1 [file DataSheet1.docx]

**SUPPORTING INFORMATION**

**Challenging DESs and ILs in the valorization of food waste: a case** **study**

Angelica Mero^1,2^, Nicholas R. Moody^3^, Elena Husanu^1^, Andrea Mezzetta^1,2^, Felicia D’Andrea^1,2^, Christian Silvio Pomelli^1,2^, Nathalie Bernaert^4^, Francesca Paradisi^3,5^, Lorenzo Guazzelli^1,2^

^1^ Department of Pharmacy, Via Bonanno 6, 56126, Università di Pisa, Italy

^2^ Consorzio INSTM, Via G. Giusti 9, 50121, Firenze, Italy

^3^Department of Chemistry, University of Nottingham, UK Department of Chemistry, University of Nottingham, UK

^4^ Flanders Research Institute for Agriculture, Fisheries and Food (ILVO), Technology and Food Science Unit, Brusselsesteenweg 370, 9090, Melle, Belgium

^5^ Department of Chemistry, Biochemistry and Pharmaceutical Sciences, University of Bern, Switzerland

**Table of content**

Volume distributions of milled fibers and residue coarse material after jet milled process page S2

FTIR and TGA of milled fibers after prewashing pages S2-S3

^1^H NMR spectra of fresh and recovered ChArg page S4

^1^H NMR spectra of fresh and recovered ChCl:LA 1:10 page S5

TGA of CRM fractions page S6

Image of CRM-ChCl:LA 120 °C page S6

FTIR and TGA comparison of CRM-ChCl:LA 90 °C and 120 °C page S7

FTIR comparison of CRM fractions and the residues obtained after enzymatic treatment page S8

TGA comparison of CRM fractions and the residues obtained after enzymatic page S9

TGA of LRM-ChArg and LRM-ChCl:LA 90 °C and 120 °C page S10

^1^H-NMR of extracted material from LRM-ChArg with hexane and DMSO page S11


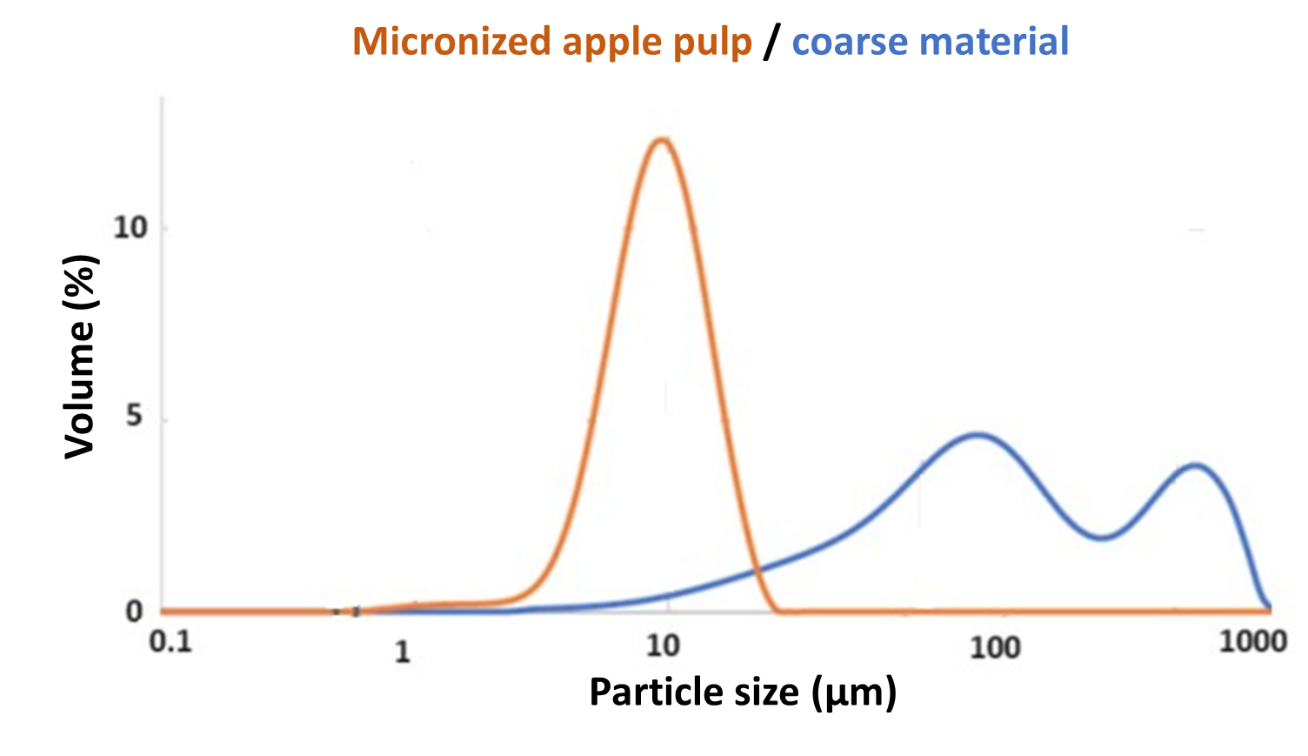


**Figure S1.** Volume distribution (distributive, top and cumulative, bottom) of milled fibers and residue coarse material after jet milled process.

**Table S1.** Volume distributions of milled fibers and residue coarse material after jet milled process.

|  | **Milled Fibers** | **Residue Coarse Material** |
| --- | --- | --- |
| **d v(10) μm** | 4.581 | 32.457 |
| **d v(50) μm** | 8.425 | 183.961 |
| **d v(90) μm** | 14.378 | 1184.205 |


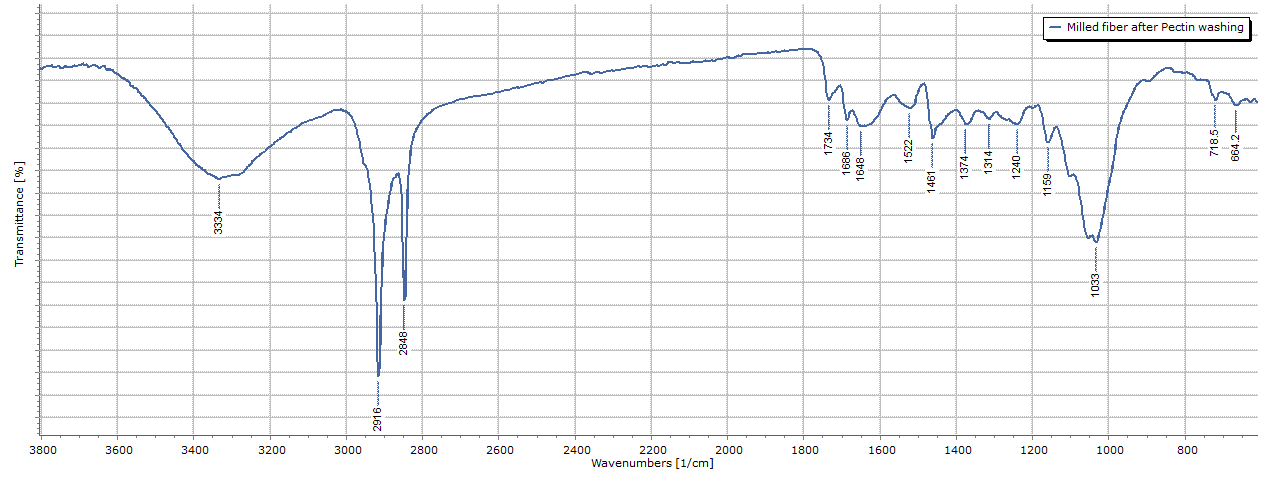


**Figure S2**. FTIR spectra of Milled Fibers after prewashing.


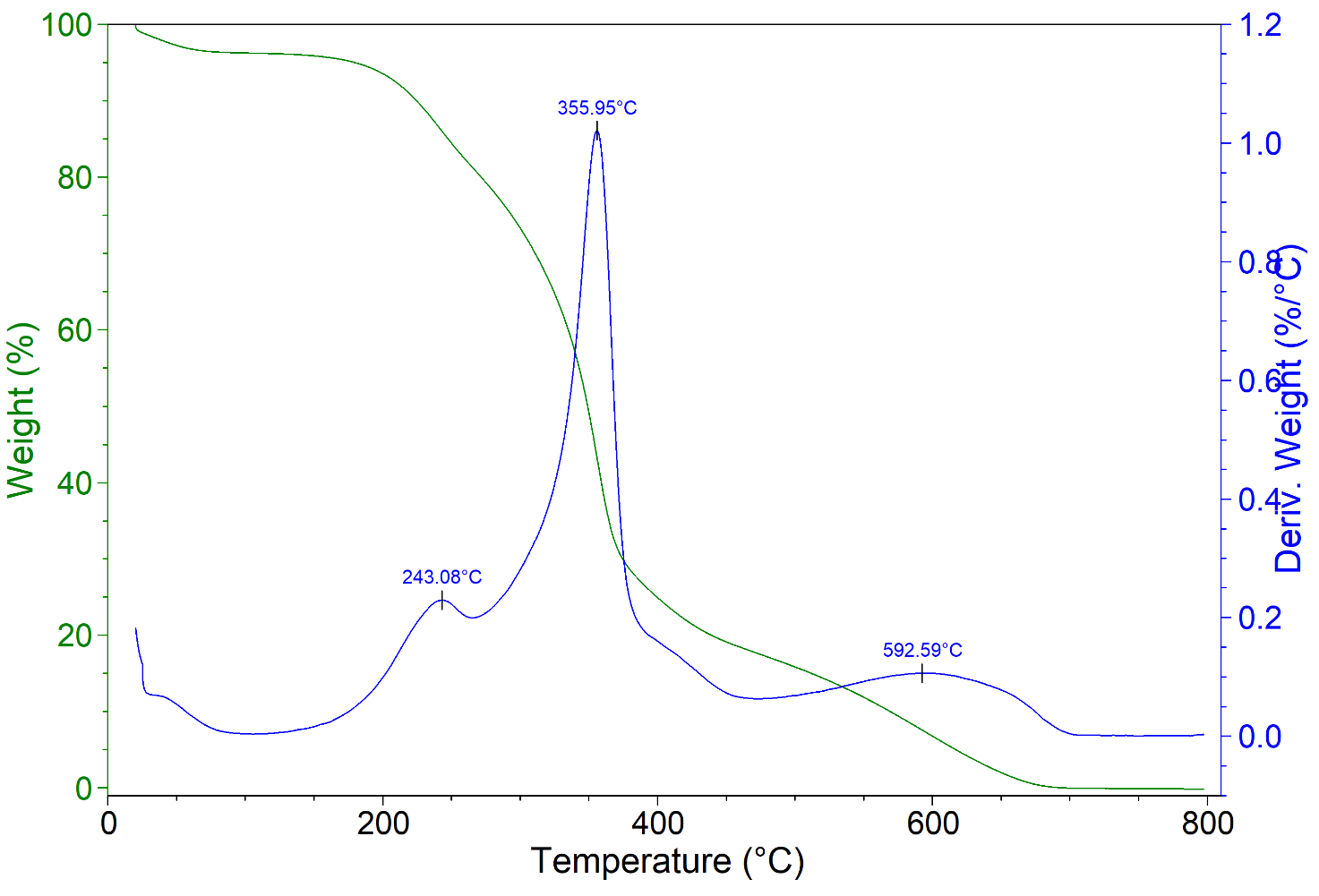


**Figure S3**. TGA of Milled Fibers after prewashing.

**Figure S4.** ^1^H-NMR comparison of fresh (top) and recovered (bottom) ChArg bio-IL.


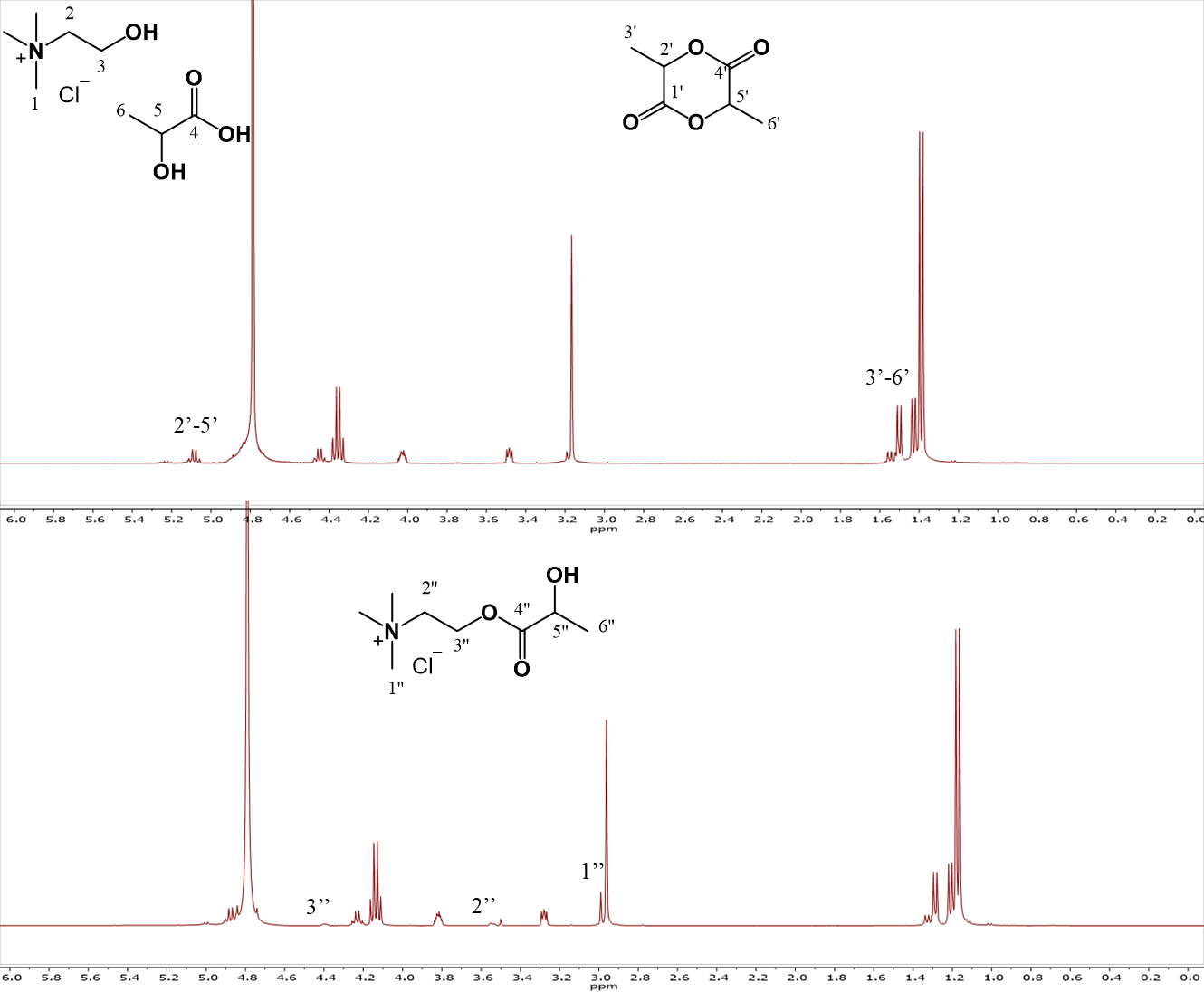


**Figure S5.** ^1^H-NMR comparison of fresh (top) and recovered (bottom) ChCl:lactic acid 1:10 NADES.


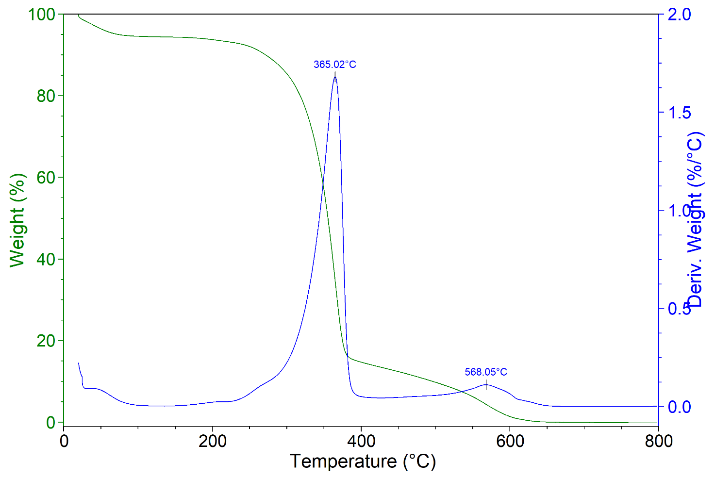

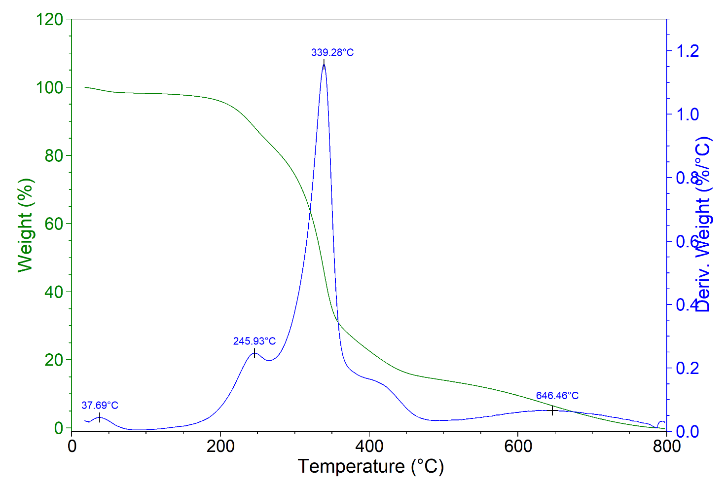


**b)**

**a)**


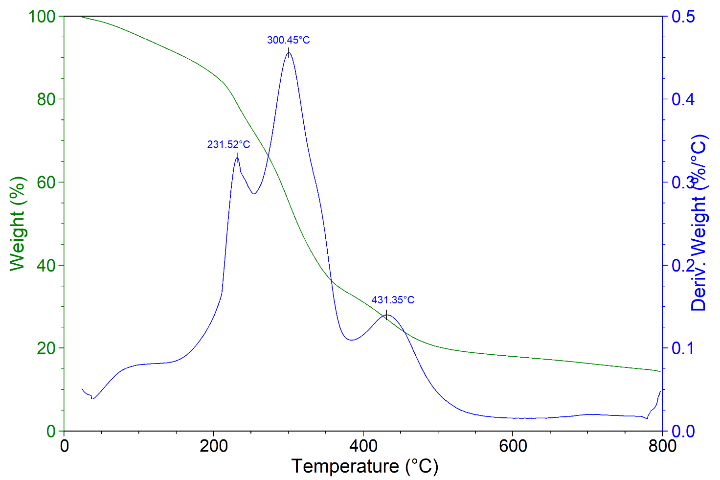

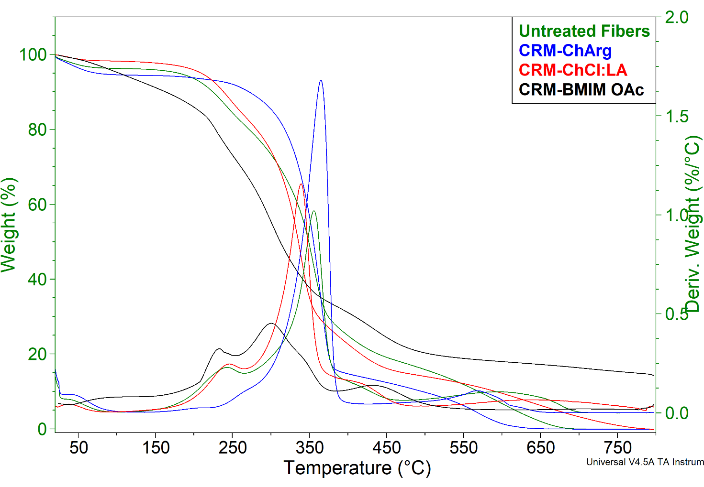


**c)**

**d)**

**Figure S6**. TGA of CRM fraction after ChArg treatment (a), CRM fraction after ChCl:LA 1:10 treatment (b), CRM fraction after BMIM OAc treatment (c), and the comparison of all the TG and DTG with that of the untreated fibers.


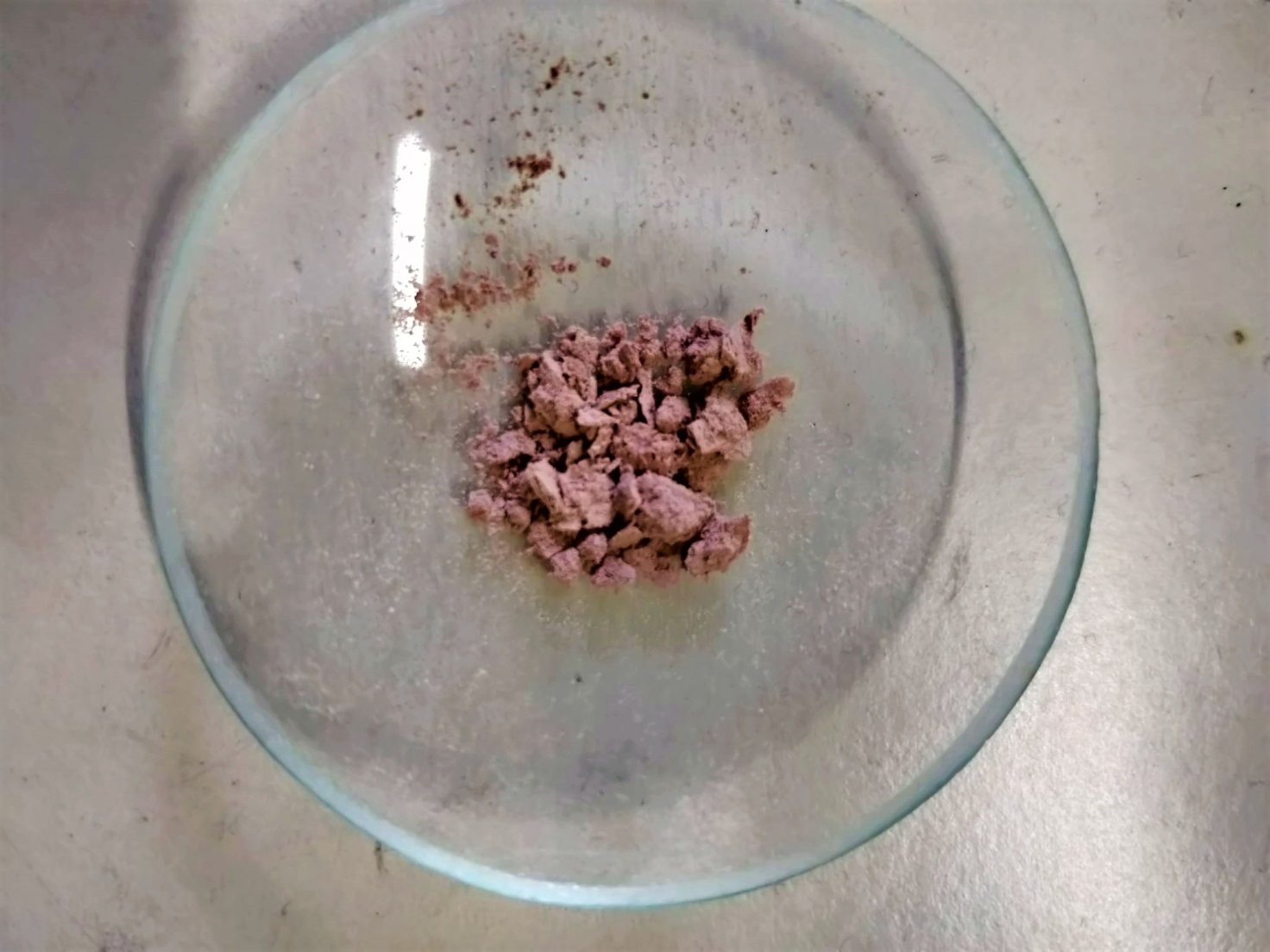


**Figure S7**. CRM from ChCl:Lactic acid 1:10 (120 °C, 1 h).

**Figure S8.** Comparison between FTIR spectra of CRM obtained with ChCl:LA treatment at 90 and 120 °C.


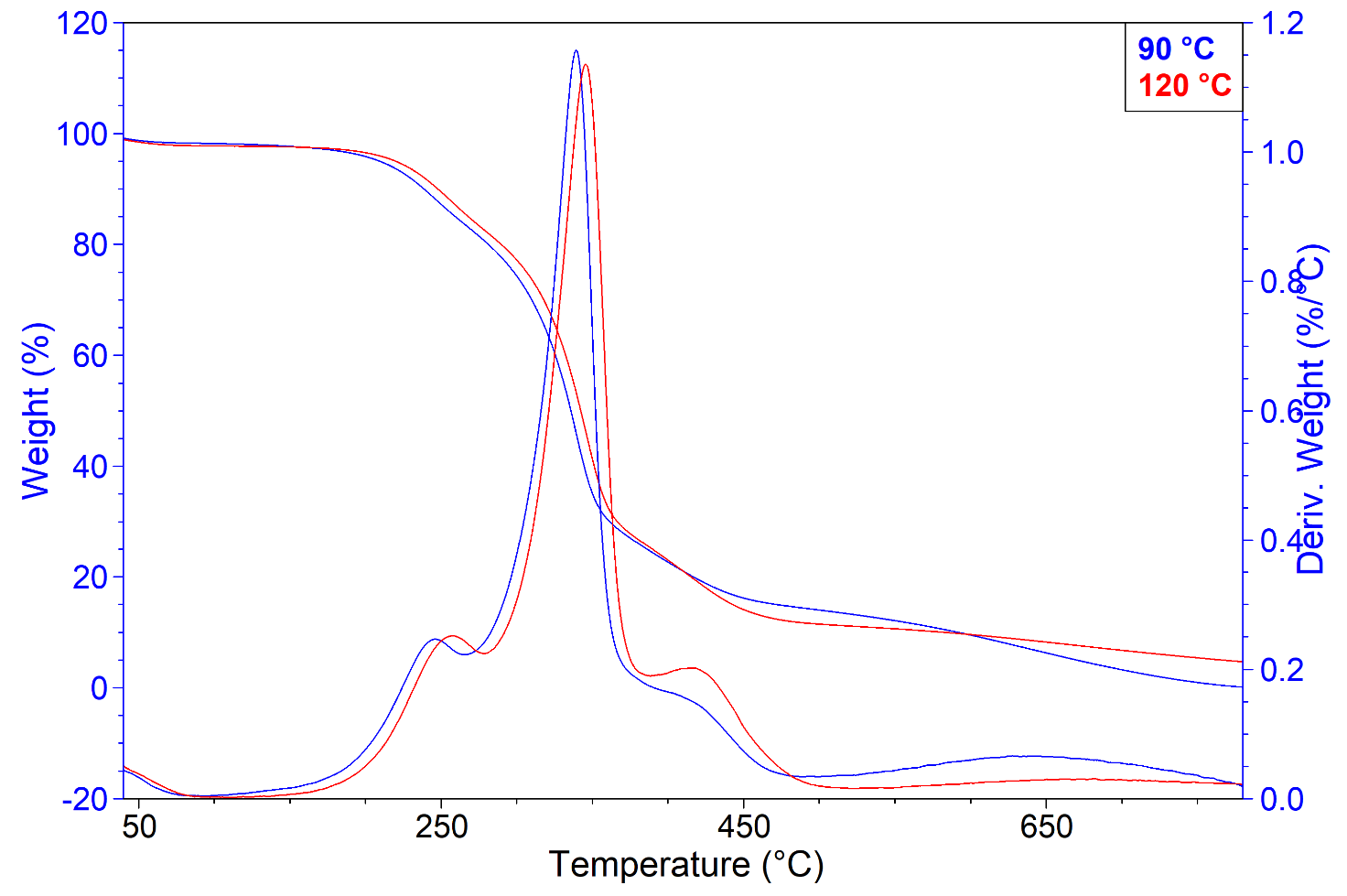


**Figure S9**. TGA of CRM fraction after ChCl:Lactic acid 1:10 treatment (90 and 120 °C, 1h).

**Figure S10.** FTIR comparison between CRM fractions and the residues obtained after enzymatic treatment.


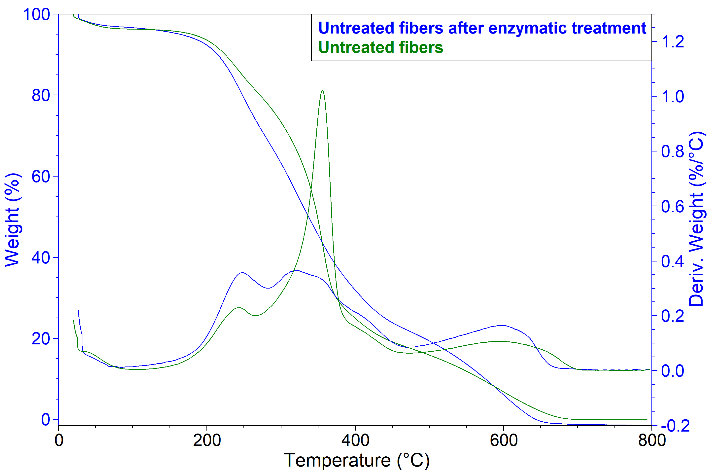

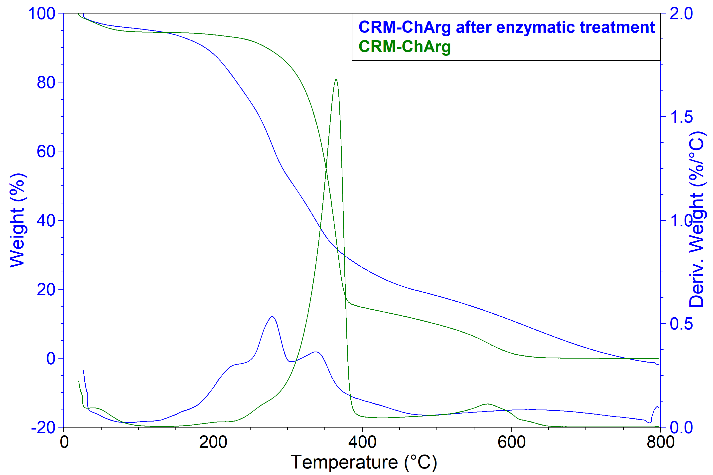


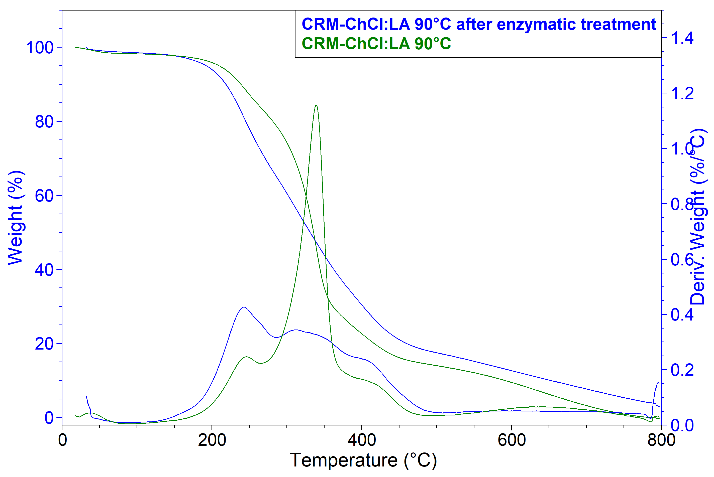

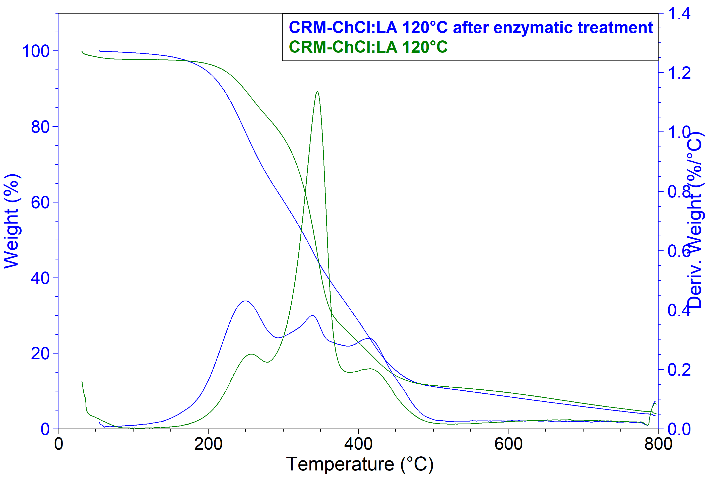


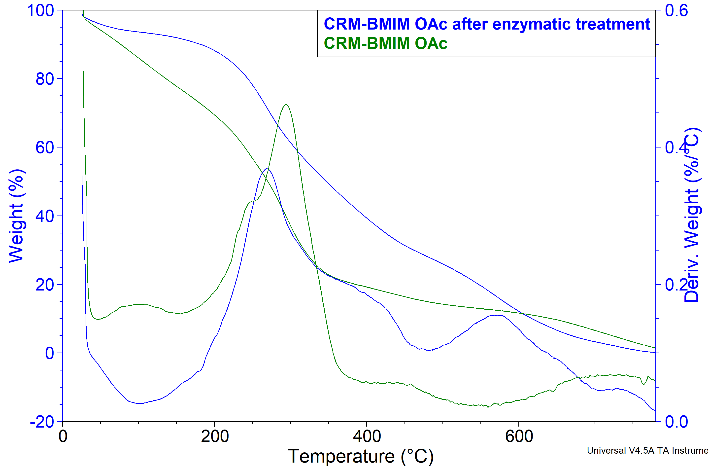


**Figure S11.** TGA comparison of CRM fractions and the residue obtained after enzymatic treatment.


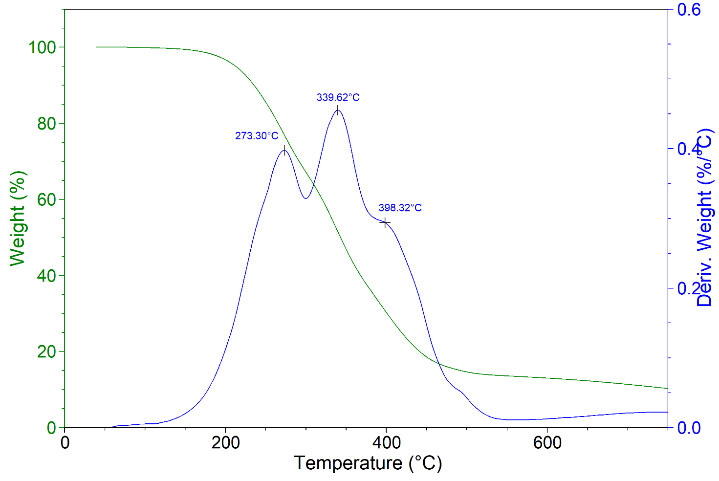

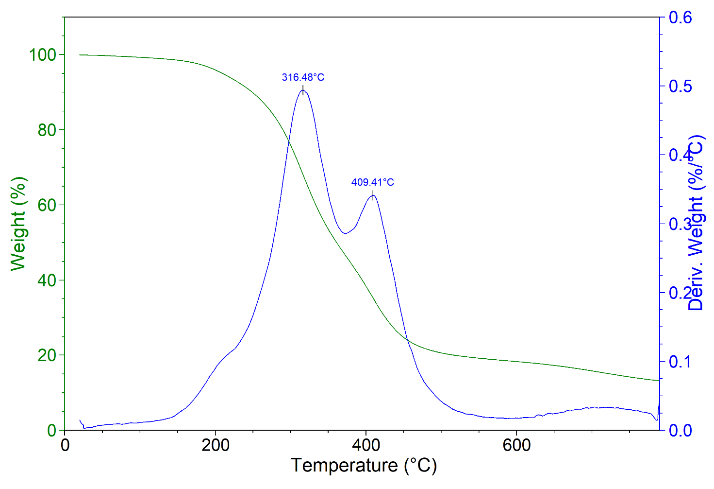


**b)**

**a)**


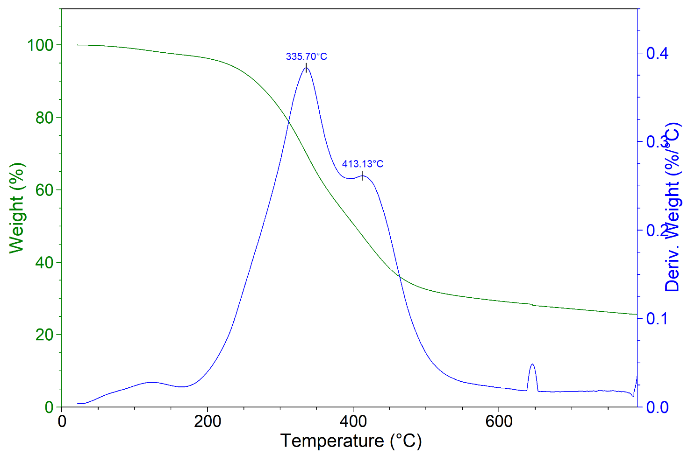


**c)**

**Figure S12**. TGA of LRM fractions obtained from pretreatment process with ChArg (a), ChCl:LA at 90 °C (b) and ChCl:LA at 120 °C (c).

**Figure S13**. FTIR spectra comparison of LRM-ChArg and the residue obtained after extraction with hexane.

**
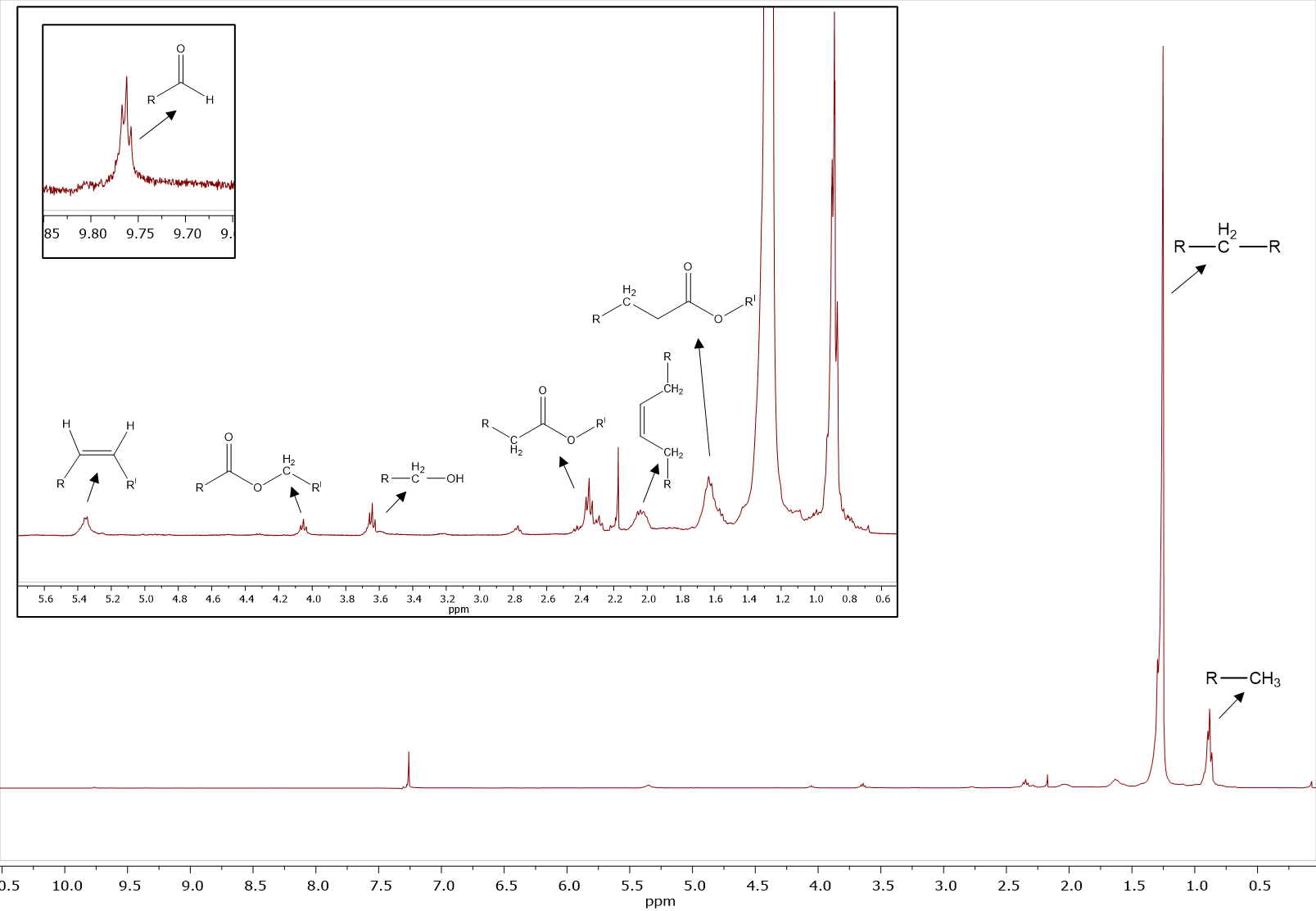
**

**Figure S14.** ^1^H-NMR of extracted material from LRM-ChArg with hexane.


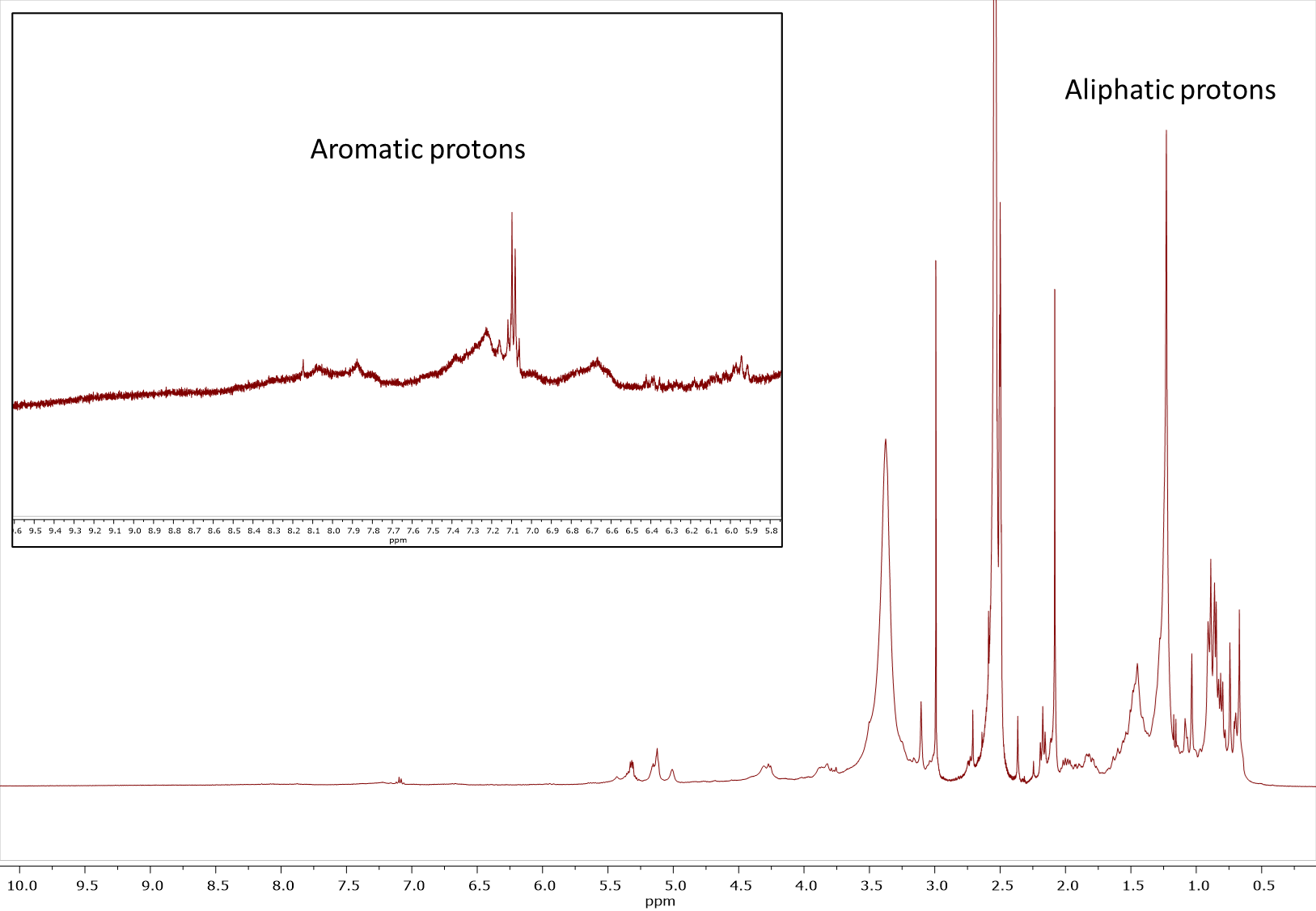


**Figure S15.** ^1^H-NMR of extracted material from LRM-ChArg with DMSO.
